# Causal Association of Coffee Consumption and Total, Knee, Hip and Self-Reported Osteoarthritis: A Mendelian Randomization Study

**DOI:** 10.3389/fendo.2021.768529

**Published:** 2021-11-10

**Authors:** Yangchang Zhang, Jun Fan, Li Chen, Yang Xiong, Tingting Wu, Shisi Shen, Xu Wang, Xuchen Meng, Yanjun Lu, Xun Lei

**Affiliations:** ^1^ School of Public Health and Management, Chongqing Medical University, Chongqing, China; ^2^ Research Center for Medicine and Social Development, Chongqing Medical University, Chongqing, China; ^3^ The Innovation Center for Social Risk Governance in Health, Chongqing Medical University, Chongqing, China; ^4^ Financial Department Chongqing Medical University, Chongqing, China; ^5^ School of Public Health & Institute of Child and Adolescent Health, Peking University, Beijing, China; ^6^ The West China Hospital, Sichuan University, Chengdu, China; ^7^ Chongqing Collaborative Innovation Center for Functional Food, Chongqing University of Education, Chongqing, China; ^8^ The First School of Clinical Medicine, Chongqing Medical University, Chongqing, China

**Keywords:** coffee consumption, osteoarthritis, SNP, Mendelian randomization, Kenn, hip

## Abstract

**Background:**

The causal association between coffee consumption and the risk of OA is limited. This study was conducted to identify the potential causal effects of coffee consumption on total, knee, hip, and self-reported OA.

**Methods:**

Genome-wide association studies (GWAS) of OA were derived from the UK Biobank, comprising 50,508 participants of European ancestry (10,083 with cases and 40,425 controls), and genetic data for specific diagnosed knee OA (4462 cases and 17,885 controls), hip OA (12,625 cases and 50,898 controls), and self-reported OA (12,658 cases and 50,898 controls). Primary and secondary genetic instruments (11 SNPs and 8 SNPs) were selected as instrumental variants from GWAS among 375,833 and 91,462 participants. Two-sample Mendelian randomization (MR) analyses were performed to test the effects of the selected single nucleotide polymorphisms (SNPs) and the OA risk. The causal effects were primarily estimated using weighted median and inverse-variance weighted method with several sensitivity analyses.

**Results:**

The MR analyses suggested that genetically predicted 1% increase of coffee consumption was associated with an increased risk of overall OA (OR:1.009, 95% CI:1.003-1.016), knee OA (OR:1.023, 95% CI:1.009-1.038), self-reported OA (OR:1.007, 95% CI:1.003-1.011), but not hip OA (OR: 1.012, 95%CI:0.999-1.024) using primary genetic instruments. Similar results were found when using secondary genetic instruments that genetically predicted coffee consumption (cups/day). Additionally, the sensitivity analyses for leave-one-out methods supported a robust association between exposure traits and OA.

**Conclusion:**

Our findings indicate that genetically predicted coffee consumption exerts a causal effect on total, knee, and self-reported OA risk, but not at the hip. Further research is required to unravel the role of coffee consumption in OA prevention.

## Introduction

Osteoarthritis (OA) is the most prevalent degenerative joint dysfunction worldwide and one of the principal causes of years lived with disability, as stated by the 2010 World Health Organization Global Burden of Disease study (1–3). This chronic disease is clinically characterized by chronic pain, crepitus, morning stiffness, and radiographic discoveries in diarthrodial joints such as the knee and hip. The incidence and prevalence of OA have risen rapidly in the past few years. The pathogenesis of OA is complex and not completely explained, but adverse lifestyles and health conditions, including excessive physical labor (4), drinking alcohol (5), smoking (6), Type 2 diabetes, and obesity (7, 8), as well as an increase of sex hormone-binding globulin (SHBG), calcium, testosterone (T) and estradiol (E2) (9), and an overburden of pathologic factors such as inflammatory cytokines and matrix degradation are known to contribute (10, 11). Additionally, previous epidemiological studies have reported an association between coffee consumption and OA risk (12). Therefore, it has aroused our interest that the causal relationship between habitual coffee consumption and the progression or suppression of OA is confirmed.

Coffee is the most popular beverage consumed in modern society and has been given substantial attention concerning its benefits and health risk (13). Coffee contains many biologically active substances like trigonelline, magnesium, potassium, niacin, lignans, heterocyclic amines, and acrylamide related to type 2 diabetes, cardiovascular disease, Alzheimer’s disease, Parkinson’s disease, cancer, and osteoarthritis (14, 15). The function of articular cartilage is dependent on the biological composition of its extracellular matrix, which comprises collagen and proteoglycans (16). In the process of OA, a gradual deterioration trend for collagens and proteoglycans is typically detected (17). A poor feature of articular cartilage is induced by prenatal caffeine exposure in male adult offspring rats (18). The molecular mechanism between caffeine intake and chondrogenesis’s retardation highlights that down-regulation of the insulin-like growth factor 1 (IGF-1) is observed in fetal growth plate cartilage in the signaling pathway (19). IGF-1 is a cardinal biomarker for keeping the function of the cartilage phenotype and cartilage anabolism (20). Therefore, an underlying causal association between coffee intake and OA risk is assumed based on these researches.

Mendelian randomization (MR) is a genetic epidemiology design, which improves the power of causal inference by applied proxies germline genetic variants as instrumental variables for exposure (e.g., coffee intake) on an outcome (e.g., OA) (21). Single nucleotide polymorphism sites (SNPs) are randomly assigned at conception, bias from reverse causation, and residual confounding is avoided (22). Although observational studies found the association between coffee intake and bone health, such as bone mass index, fracture, rheumatoid arthritis, and osteoarthritis (12, 23, 24), data are limited for the relationship of genetically predicted habitual coffee intake concerning the bone disease. One MR analysis support that coffee intake is causally associated with an increased risk of OA (25). However, the study is limited to the unclear selection and less quantity for SNPs. The causal evidence cannot be ruled out due to low statistical power, pleiotropy, and collider bias (14).

The purpose of the current study was to explore whether there was a causal relationship between coffee consumption and OA by a two-sample MR analysis.

## Methods

### Genetic Instrument Selection

The European population’s genome-wide association meta-analysis (GWAS) dataset identified SNPs associated with coffee consumption. As for primary genetic instruments, 15 significant SNPs associated with coffee intake were obtained from a GWAS meta-analysis that comprised over 370,000 participants of European ancestry (25). This GWAS adjusted for age, sex, BMI, total energy, the proportion of 24 hours recalls self-reported as capturing “typical intake,” and top twenty principal components (25). Habitual coffee intake was retrieved based on the item: “How many cups of coffee do you drink each day (include decaffeinated coffee)? “ Meanwhile, the effects of SNPs were interpreted as 1% change of coffee consumption per effect allele (25). As for secondary genetic instruments, 10 significant SNPs associated with the cups of coffee consumed per day were identified from another GWAS, including 91,462 participants released by the Coffee and Caffeine Genetics Consortium (CCGC) (26). All instrumental variables were associated with the exposure (e.g., coffee consumption) at a genome-wide significance level (*P*<5×10^-8^) with linkage disequilibrium (LD) *r^2^* <0.001 at a 10,000 kb window, which confirmed the independence for the selected genetic variants. In this MR study, 11 and 8 independent SNPs with moderate LD were selected as genetic instruments for habitual coffee consumption after excluding four SNPs (rs117692895, rs4719479, rs12699844, and rs73073176 in chromosome 7) and two SNPs (rs6968554 in chromosome 7; rs247083 in chromosome 15) in primary genetic instruments and secondary genetic instruments, respectively. Detailed information on the relationship between the selected SNPs and exposures is shown in **Table 1**.

**Table 1:** Characteristics of SNPs for habitual coffee consumption from the GWAS meta-analysis.

GWAS	chr	Pos	SNP	Closest gene	EA	OA	EAF	Effect	SE	*P* value	N
Primary (% change)	1	177873210	rs574367	SEC16B	T	G	0.21	1.05	0.18	8.E-09	376372
Primary	2	631606	rs10865548	TMEM18	G	A	0.83	1.54	0.19	4.46E-15	376372
Primary	2	27730940	rs1260326	GCKR	C	T	0.61	1.36	0.15	2.62E-19	376372
Primary	7	17284577	rs4410790	AHR	C	T	0.63	3.94	0.15	5.59E-141	376372
Primary	7	73037956	rs34060476	MLXIPL	G	A	0.13	1.89	0.22	5.06E-18	376372
Primary	7	75615006	rs1057868	POR	T	C	0.29	1.97	0.16	5.26E-33	376372
Primary	11	56272114	rs597045	OR8U8	A	T	0.69	1.07	0.16	6.62E-11	376372
Primary	14	33075243	rs1956218	AKAP6	G	A	0.56	0.82	0.15	3.62E-08	376372
Primary	15	75027880	rs2472297	CYP1A1/2	T	C	0.27	4.54	0.17	5.19E-155	376372
Primary	18	57808978	rs66723169	MC4R	A	C	0.23	1.47	0.18	9.88E-17	376372
Primary	22	24747031	rs2330783	SPECC1L-ADORA2A	G	T	0.99	4.53	0.63	1.57E-12	376372
Secondary (Cups/day)	2	27584444	rs1260326	GCKR	T	C	0.41	-0.04	0.01	1.06E-07	91462
Secondary	4	89258106	rs1481012	ABCG2	A	G	0.89	0.06	0.01	1.13E-06	91462
Secondary	7	17251102	rs4410790	AHR	T	C	0.37	-0.14	0.01	1.48E-57	91462
Secondary	7	72673793	rs7800944	MLXIPL	T	C	0.72	-0.05	0.01	7.82E-09	91462
Secondary	7	75454041	rs17685	POR	A	G	0.29	0.07	0.01	9.06E-14	91462
Secondary	11	27636492	rs6265	BDNF	T	C	0.19	-0.05	0.01	3.40E-07	91462
Secondary	15	72814933	rs2472297	CYP1A2	T	C	0.24	0.15	0.01	6.45E-47	91462
Secondary	17	25373221	rs9902453	EFCAB5	A	G	0.54	-0.04	0.01	2.26E-06	91462

Chr, chromosome; Pos, position for SNP; Closet gene, the nearest gene to coffee consumption associated SNP; Effect, the per-allele effect on coffee consumption; P value, the value for the genetic association; GWAS, genome-wide association study; SNP, single-nucleotide polymorphism; EA, effect allele; OA, other allele; EAF, effect allele frequency; SE, standard error.

### Genetic Summary Data of Osteoarthritis

The primary outcome in this study was the clinically diagnosed OA. The summary-level data for OA were obtained from the large GWAS conducted by the UK Biobank, enrolling 50,508 participants of European ancestry (10,083 with cases and 40,425 controls). Moreover, the secondary outcomes were specific diagnosed knee OA (4462 cases and 17,885 controls) and hip OA (12,625 cases and 50,898 controls), and self-reported OA (12,658 cases and 50,898 controls). Study protocols related to these data have been released and described in the previous studies (27–29). All summary data can be obtained from the UK Medical Research Council Integrative Epidemiology Unit Open GWAS Project database (httep://gwas.mrcieu.au.uk). The relevant ethics committees approved all studies that contributed data to these analyses, and all participants provided written informed consent.

### Statistical Analysis

The conventional MR method was applied in this study (https://mrcieu.github.io/TwoSampleMR/articles/perform_mr.html#mr-methods-1) (30). The random-effects inverse-variance weighted (IVW) model was conducted to examine the causal association, and this approach was considered as the main analysis because of the potential observed heterogeneity (31, 32). The IVW method combines individual MR effects across SNPs to derive an overall weighted effects of the potential causal association. Furthermore, the forest plots were showed to visualize the MR-derived odds ratio (OR) of overall, knee, hip, and self-reported OA risk for 1% or one cup per day increase in genetically predicted coffee intake. In the sensitivity analyses, “leave-one-out” method was performed to estimate that the causal association was reliant on any single SNP. In addition, Steiger-MR was used to test whether the SNPs explained significantly more variance in exposure than outcome (the opposite may indicate reverse causation).

The IVW method assumes that all genetic variants should satisfy three assumptions for the instrumental variables. 1) strongly associated with coffee intake, 2) not associated with confounders of the association between coffee intake and OA, and 3) the association with OA risk was only found *via* coffee consumption (33, 34). Thus, the F statistics were used to test for weak instrumental variables. F statistics=((n-k-1)/k)(R^2/(1-R^2)), where R^2^ is the variance in coffee consumption explained by the genetic instrument, *k* is the number of genetic variants, and n is the sample size. The strengths of the primary (11 SNPs) and Secondary (8 SNPs) genetic instruments used for analyses were 70.59 and 28.46, respectively. *F* > 10 was proven to use strong genetic instruments in MR study. The Cochran’s Q test was used to quantify the heterogeneity in effect sizes between the genetic instruments (35), which may indicate horizontal pleiotropy that could violate the third MR assumption. Potential violation of the second and third MR assumptions was tested using several approaches such as the MR-Egger regression (36), the weighted median (37) and mode (38) methods, and the MR pleiotropy residual sum and outlier test (MR-PRESSO) (39). The association between the selected SNPs and exposures was validated in the PhenoScanner database (http://www.phenoscanner.medschl.cam.ac.uk/) (**Supplementary Table 1**). SNP associated with traits other than coffee consumption at the GWAS significance level was documented.

The MR-Egger approach is an adaption for Egger regression that allows for directional pleiotropy by introducing an intercept in the weighted regression model. Horizontal pleiotropy was indicated when values were away from zero for the intercept term (36). Based on this approach, unbiased estimates are performed in the presence of pleiotropic instruments assuming that the magnitude of pleiotropic effects is independent of the size of the instrumental variables—SNPs associated with coffee intake (36).

Weighted median method orders the estimates in MR using each instrument weighted for the inverse of their variance, and the median result is selected and shows the single MR estimate with confidence intervals based on bootstrapping technique (37). The weighted median requires and assumes that at least half of the instruments are valid (40).

The mode-based causal estimate consistently estimates the true causal effect when the most instruments with consistent MR estimates are valid (38).

The “twosampleMR” package (version 0.5.5) and R software version 3.6.1 were used for all statistical analyses. Bonferroni correction (P = 0.05/2 exposures/4 outcomes = 0.0625) was implemented for multiple comparisons. Two-sided P values were computed, with *P* < 0.00625 regarded as statistically significant.

## Results

### Causal Associations Between Coffee Consumption and Overall OA

**Figure 1:**
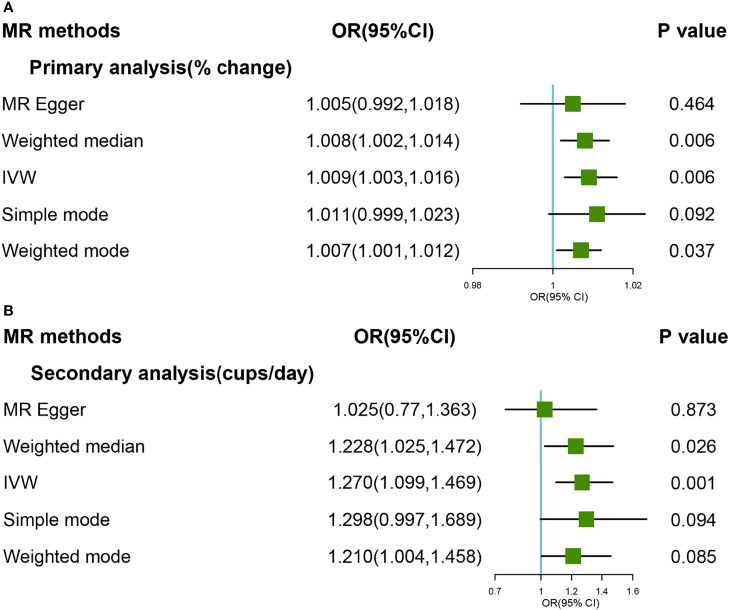
Forest plot of MR study using **(A)>** primary genetic instruments with total OA. MR, Mendelian randomization; OR, odds ratio; CI, confidence interval; IVW, inverse variance weighted; OA, Osteoarthritis. Forest plot of MR study using **(B)** secondary genetic instruments with total OA. MR, Mendelian randomization; OR, odds ratio; CI, confidence interval; IVW, inverse variance weighted; OA, Osteoarthritis.

The primary and secondary genetic instruments using IVW with random effect analyses provide strong evidence for the causal association between genetically predicted coffee consumption and overall OA (OR:1.009, 95%CI:1.003-1.016; OR:1.270, 95%CI:1.099-1.469) (**Figures 1A**, **B**). The estimates for each SNP on overall OA were shown in **Supplementary Figures 1**, **2**. The scatter plot for two analyses was attached as **Supplementary Figures 3**, **4**.

As for primary genetics instruments, Cochran’s Q statistic suggested a slight sign of heterogeneity: Q value (df)=18.34 (9), *P=*0.03 for MR Egger method; Q value (df)= 19.56 (10), *P*=0.03 for IVW method. However, the significant effect was support for Weight Median methods that can be given priority when the model with heterogeneity but no pleiotropy (OR:1.008, 95%CI:1.002-1.014). In addition, there was no indication of pleiotropy when the intercept was derived from the MR-Egger regression (Egger intercept:0.01, *P* value:0.46). Furthermore, no outliers were detected with potential pleiotropy using the MR-PRESSO method. Moreover, the *P*-value for the MR-PRESSO Global test was 0.073, which also suggested no significant sign of heterogeneity. The leave-one-out analysis results suggested no influential SNPs from the causal link between coffee intake and OA in the replication analyses (**Supplementary Figure 5**). Steiger filtering identified 2 instruments explaining more variation in total OA than in caffe consumption. Removing those slightly attenuated the identified effect estimate, which was still suggestive of a significant effect of genetic liability to higher cups of coffee intake per day on risk of total OA in the primary analyses (**Supplementary Table 2**).

In the secondary analyses, Cochran’s Q statistic suggested no sign of heterogeneity: Q value (df)=4.50 (6), *P*=0.61 for MR-Egger method; Q value (df)= 7.39 (7), *P*=0.38 for IVW method. The associations were consistent with using the weighted median method (OR = 1.228, 95% CI: 1.025–1.472; *P* = 0.026). In addition, there was no indication of pleiotropy when the intercept was derived from the MR-Egger regression (Egger intercept:0.02, *P* value:0.14). Furthermore, no outliers were detected with potential pleiotropy using the MR-PRESSO method. The leave-one-out analysis results suggested no influential SNPs from the causal link between coffee intake and OA in the replication analyses (**Supplementary Figure 6**). Steiger filtering suggested that the direction of the effect was correct for all the coffee consumption instruments in the secondary analyses.

### Causal Associations Between Coffee Consumption and Knee OA

**Figure 2:**
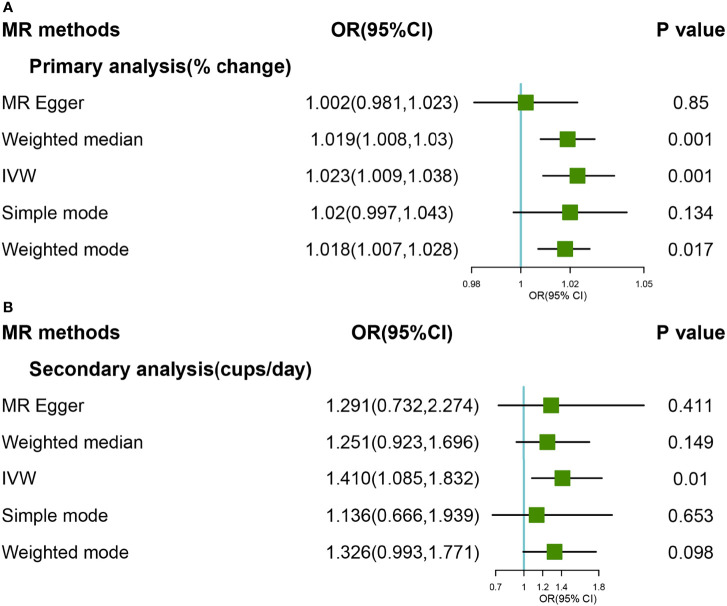
Forest plot of MR study using **(A)** primary genetic instruments with knee OA after excluding four SNPs (rs4410790, rs597045, rs34060476, and rs1956218). MR, Mendelian randomization; OR, odds ratio; CI, confidence interval; IVW, inverse variance weighted; OA, Osteoarthritis. Forest plot of MR study using **(B)** secondary genetic instruments with knee OA. MR, Mendelian randomization; OR, odds ratio; CI, confidence interval; IVW, inverse variance weighted; OA, Osteoarthritis.

As for primary genetic instruments, IVW with random effect suggested marginally significant evidence for the causal association between coffee consumption and OA (OR:1.01,95% CI:0.999-1.025). However, strong heterogeneity was found in both MR-Egger (Q value:32.81, *P* value<0.0001) and IVW methods (Q value: 32.96, P value<0.0001), respectively. The hypothesis of the pleiotropy test was satisfied in the fitting model. By MR PRESSO method, one SNP (rs34060476) was detected as an outlier, and the Global test was 0.012. Based on leave-one-out methods, we found 4 SNPs (rs4410790, rs597045, rs34060476, and rs1956218) that were potential sources of heterogeneity (**Supplementary Figure 11** **).** After excluding these SNPs (rs4410790, rs597045, rs34060476 and rs1956218), results from the Weight Median and IVW methods suggested a strong causal association (OR:1.019,95%CI:1.008-1.030; OR:1.023, 95%CI:1.009-1.038) (**Figure 2A**). The forest and scatter plots were performed in **Supplementary Figures 7**, **9**, respectively.

In the secondary analyses, the causal relationship was supported for the IVW method (OR:1.410, 95% CI:1.085-1.832), and The effect was slightly attenuated with the Weight Median method, yielding a null causal estimation between coffee consumption and OA (OR:1.251, 95% CI: 0.923-1.696) (**Figure 2B**). The forest and scatter plots were performed in **Supplementary Figures 8**, **10**, respectively. There was no heterogeneity and outlier in the model (*P >*0.05). The leave-one-out analyses revealed that the result was essentially consistent after removing SNP in turn (**Supplementary Figure 12** **).**

Steiger filtering suggested that 3 and 1 SNPs associated with knee OA explained more variation in coffee consumption and these were removed in primary and secondary dataset, respectively. The exclusion of these SNPs, despite attenuating the two analyses effect estimate, were suggestive of an effect of genetic liability to higher coffee consumption on knee OA (**Supplementary Table 2**).

### Causal Associations Between Coffee Consumption and Hip OA

**Figure 3:**
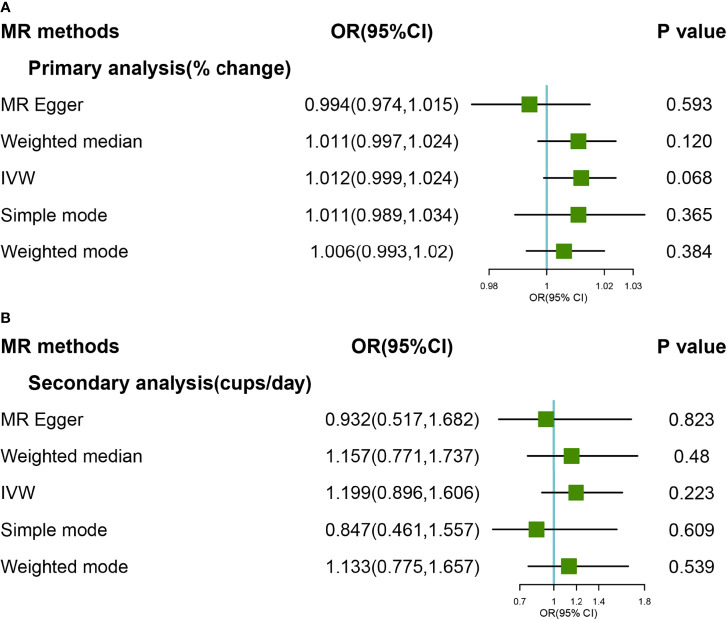
Forest plot of MR study using **(A)** primary genetic instruments with hip OA. MR, Mendelian randomization; OR, odds ratio; CI, confidence interval; IVW, inverse variance weighted; OA, Osteoarthritis. Forest plot of MR study using **(B)** secondary genetic instruments with hip OA. MR, Mendelian randomization; OR, odds ratio; CI, confidence interval; IVW, inverse variance weighted; OA, Osteoarthritis.

The causal association between coffee consumption and hip OA was not found based on IVW analyses, either primary or secondary genetic instruments (OR:1.012, 95% CI: 0.999-1.024; OR: 1.199, 95% CI: 0.896-1.606) (**Figures 3A**, **B**). The hypothesis of both heterogeneity and pleiotropy was testified (*P >*0.05). The forest and scatter plots were performed in **Supplementary Figures 13**–**16**, and the plot for leave-one-out analyses was shown in **Supplementary Figures 17**, **18**. Steiger filtering which explain more variation in the outcome across two analyses, the effect estimate attenuated further, providing limited evidence of a direct effect of genetic liability to higher coffee consumption on the risk of hip OA (**Supplementary Table 2**).

### Causal Associations between Coffee consumption and Self-reported OA

**Figure 4:**
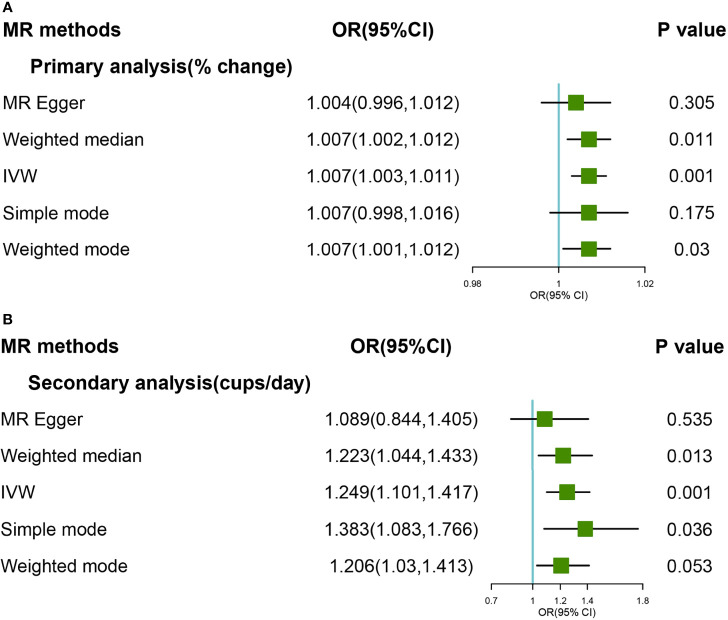
Forest plot of MR study using **(A)** primary genetic instruments with self-reported OA. MR, Mendelian randomization; OR, odds ratio; CI, confidence interval; IVW, inverse variance weighted; OA, Osteoarthritis. Forest plot of MR study using **(B)** secondary genetic instruments with self-reported OA. MR, Mendelian randomization; OR, odds ratio; CI, confidence interval; IVW, inverse variance weighted; OA, Osteoarthritis.

A significant causal relationship was found between genetically predicted coffee consumption and self-reported OA in primary and secondary genetic instruments (**Figures 4A**, **B** **)**. In primary analyses, the OR for OA was 1.007 (95% CI:1.003-1.011) using the IVW method and 1.007 (95% CI:1.002-1.012) using the Weight Median method; In secondary analyses, the OR for OA was 1.249 (95%CI:1.101-1.417) using the IVW method and 1.223 (95% CI:1.044-1.433) using weighted median. There was no significant heterogeneity and pleiotropy (*P >*0.05). None outlier was confirmed in MR PRESSO analyses. Detailed forest and scatter plots were shown in **Supplementary Figures 19**–**22**. The plot for leave-one-out analyses was shown in **Supplementary Figures 23**, **24**.

Steiger filtering suggested that the effect direction was correct for all the coffee consumption SNPs, and no SNP was removal (**Supplementary Table 2**).

## Discussion

This conventional MR study used summary-level data from two large GWAS datasets and UK Biobank to estimate the potential causal association between coffee intake and OA types. Based on the primary and secondary genetic instruments results, coffee consumption was causally associated with increased risk of OA, including total OA and self-reported OA. Habitual coffee consumption was shown to be causal for knee OA, but not hip OA. Therefore, the most novel finding from this study is that coffee consumption is a potential risk for OA, and this causal effect was different with specific join sites. This causal association is free of reverse causation, selection bias, and sample sizes.

Few epidemiological studies are related to the association between coffee consumption and OA. In the cross-sectional study from the Korea National Health and Nutrition Examination Survey, daily coffee drinking more than 7 cups were associated with the high risk of OA in Korean men. This association was a linear trend with increasing coffee consumption (12). However, in the scope of clinic medicine or basic experiment, much data points to the effective negative impacts of caffeine consumption on hyaline cartilage (30). The adverse effects of caffeine on the articular cartilage were reported in rat models, and low-dose prenatal caffeine exposure (PCE) significantly affected fetal articular integrity in some pregnant women (19, 41, 42). Previous studies revealed that rat offspring with PCE had irregular surface cartilage with uneven and altered chondrocytes in the tangential zone (18). Additionally, caffeine directly affects chondrocytes by inhibiting adenosine receptors, well-known as anti-inflammatory and anabolic effects on chondrocytes and other articular cells (43, 44). Finally, indirect effects of caffeine between inflammatory factors and articular cartilage have been proposed in previous studies, and caffeine consumption was associated with the inflammatory cytokine IL-1 and TNF-α (45).

Two MR studies in the past reported the causal association between coffee consumption and risk of OA. In the one MR study, the GWAS meta-analyses were consistent of eight Caucasian cohorts (18,176) and European ancestry (91,462), which revealed a positive causal association between coffee consumption and OA (46). However, fewer power samples, a small number of SNPs, weak instrument variants, and an unclear process cannot be completely demonstrated. In another Mendelian randomization phenome-wide association study (MR-PheWAS), the causal association between the full range of disease outcome and instrumented habitual coffee consumption were examined with 333,214 participants of White-British ancestry in the UK Biobank and found the causal relationship between consumption coffee and total OA (47). Nevertheless, it is unclear how the selected SNPs satisfy the hypothesis of linkage disequilibrium, strong instrument variants, statistic power, and discharge of covariates. Significantly, the single type of outcome or exposure may increase the probability of false positives. In our study, four types of outcome were identified in the MR study, such as total OA, knee OA, hip OA and self-reported OA, which increased the robustness results. We also found the modification of coffee consumption on different joint sites (knee and hip).

MR method is less likely to possess bias from humans compared to retrospective analyses and case-control prospective studies. The frequency and amount of coffee consumption are often imprecise in a survey, and recall bias is unavoidable in the observational study. Additionally, data collection and analysis can be costly; the MR methods can mitigate this issue to some extent; sequential application of different algorithms in sensitivity analyses can improve the accuracy and reliability of results. In our study, five MR algorithms were used to estimate this causal association, including IVW, MR-Egger, weighted median, weighted mode, and MR-PRESSO. In this study, the results from the IVW (Primary: OR=1.009, *P*<0.01; Secondary: OR=1.27, *P*<0.001) and weighted median (Primary: OR=1.008, *P*<0.01, Secondary: OR=1.22, *P*<0.05) methods supported an inverse causative association between coffee consumption and OA. However, the MR-Egger method revealed no causal association between coffee consumption and OA (Primary: OR=1.004, *P*=0.46; Secondary: OR=1.02, *P*= 0.87). Based on previous studies, the IVW and weighted median algorithms were superior to the MR-Egger, which estimates that both the intercept and the slope with only five instrumental variants are not robust enough (48).

This study has several key strengths. Firstly, a large sample size with summary-level genetic data was available in this study, and replicative analyses (Primary and Secondary genetic instruments) were used to improve the study’s credibility. Secondly, the MR-PRSSO method was conducted to find outliers and demonstrate the pleiotropy test. Thirdly, we replicated our results by using the different definitions and joint sites for OA and found similar results and modifications on specific joints. Lastly, the “leave-one-out” method was performed as sensitivity analyses increased the robustness of the results.

There were some limitations in this study. Firstly, the results were not applicable to other populations due to the potential biased that the data only derived from European populations. Secondly, any nonlinear relationships or stratification effects were not conducted because of the summary-level data. Lastly, the type of coffee beans, the amount of intake, the ways of roasting and brewing were important for exploring the causal association on exposure, and further study should consider the effect of coffee consumption as a whole.

## Conclusion

In conclusion, there was a positive causal association between coffee consumption and total OA and self-reported OA, and this major causal effect was found at knee OA but not at the hip. This MR study was a complete and detailed analysis of coffee consumption and OA, which provided new evidence for prevention or therapeutic strategies for the different OA sites.

## Data Availability Statement

The datasets presented in this study can be found in online repositories. The names of the repository/repositories and accession number(s) can be found in the article/**Supplementary Material**.

## Ethics Statement

Ethical review and approval were waived for this study, all the data from Mendelian randomization is publicly accessible. Informed consent was obtained from all subjects in the original genome-wide association studies. The patients/participants provided their written informed consent to participate in this study.

## Author Contributions

YZ, YX, LC, TW, SS, YL, and XL designed the study. YX analyzed the data. YZ wrote the manuscript. All authors contributed to the article and approved the submitted version.

## Funding

This work was supported by the National Natural Science Foundation of China (Grant No. 72174033) and the Natural Science Foundation General Project of Chongqing Science and Technology Bureau (Grant No. cstc2020jcyj-msxmX0279).

## Conflict of Interest

The authors declare that the research was conducted in the absence of any commercial or financial relationships that could be construed as a potential conflict of interest.

## Publisher’s Note

All claims expressed in this article are solely those of the authors and do not necessarily represent those of their affiliated organizations, or those of the publisher, the editors and the reviewers. Any product that may be evaluated in this article, or claim that may be made by its manufacturer, is not guaranteed or endorsed by the publisher.
