# Supplementary material for: Causal Association of Coffee Consumption and Total, Knee, Hip and Self-Reported Osteoarthritis: A Mendelian Randomization Study
Source: Front Endocrinol (Lausanne). 2021 Nov 10;12:768529. doi: 10.3389/fendo.2021.768529 (PMC8631393; doi:10.3389/fendo.2021.768529)
Supplement: Supplementary file 1 [file DataSheet_1.docx]

Supplementary Material

**Supplementary Tables**

**Table 1S.** Potential confounders associated with selected SNPs.

| Primary | rs574367 | Age at menarche | 8.E-09 |
| --- | --- | --- | --- |
|  | rs10865548 | Age at menarche | 4.46E-15 |
|  | rs1260326 | Triglycerides;C reactive protein;Cholesterol;drinking;Monocyte count;Neutrophil count; Lymphocyte count;Type II diabetes;Age at menopause | 2.62E-19 |
|  | rs117692895 | / | 4.13E-10 |
|  | rs4410790 | / | 5.59E-141 |
|  | rs4719497 | / | 4.23E-08 |
|  | rs12699844 | / | 1.35E-16 |
|  | rs73073176 | / | 5.06E-25 |
|  | rs34060476 | Triglycerides Monocyte count | 5.06E-18 |
|  | rs1057868 | / | 5.26E-33 |
|  | rs597045 | / | 6.62E-11 |
|  | rs1956218 | / | 3.62E-08 |
|  | rs2472297 | / | 5.19E-155 |
|  | rs66723169 | drinking | 9.88E-17 |
|  | rs2330783 | / | 1.57E-12 |
| Secondary | rs1260326 | Triglycerides;C reactive protein;Cholesterol;drinking;Monocyte count;Neutrophil count; Lymphocyte count;Type II diabetes;Age at menopause | 1.06E-07 |
|  | rs1481012 | LDL cholesterol | 1.13E-06 |
|  | rs4410790 | / | 1.48E-57 |
|  | rs6968554 | / | 2.54E-57 |
|  | rs7800944 | Triglycerides;Monocyte count | 7.82E-09 |
|  | rs17685 | / | 9.06E-14 |
|  | rs6265 | Age at menarche;smoking;Falls in the last year; | 3.40E-07 |
|  | rs2470893 | / | 6.89E-44 |
|  | rs2472297 | / | 6.45E-47 |
|  | rs9902453 | Monocyte count;Heel bone mineral density | 2.26E-06 |

**Table 2S.** Causal effect estimates of genetic predicted coffee consumption on risk of OA after removing instruments identified to explain more variation in the outcome through Steiger filtering.

| Instrumental variables | Traits | N.SNP | OR | 95% CI | *P* |
| --- | --- | --- | --- | --- | --- |
| Primary SNPs | Total OA | 9 | 1.007 | 1.002-1.013 | 0.0067 |
|  | Knee OA | 4 | 1.016 | 1.007-1.027 | 0.0007 |
|  | Hip OA | 9 | 1.008 | 0.997-1.017 | 0.122 |
|  | Self-reported OA | - | - | - | - |
| Secondary SNPs | Total OA | - | - | - | - |
|  | Knee OA | 7 | 1.359 | 1.072-1.722 | 0.010 |
|  | Hip OA | 7 | 1.157 | 0.859-1.557 | 0.335 |
|  | Self-reported OA | - | - | - | - |

*Note.* The Odds Ratio was obtained from IVW method.

**2 Supplementary Figures**

**
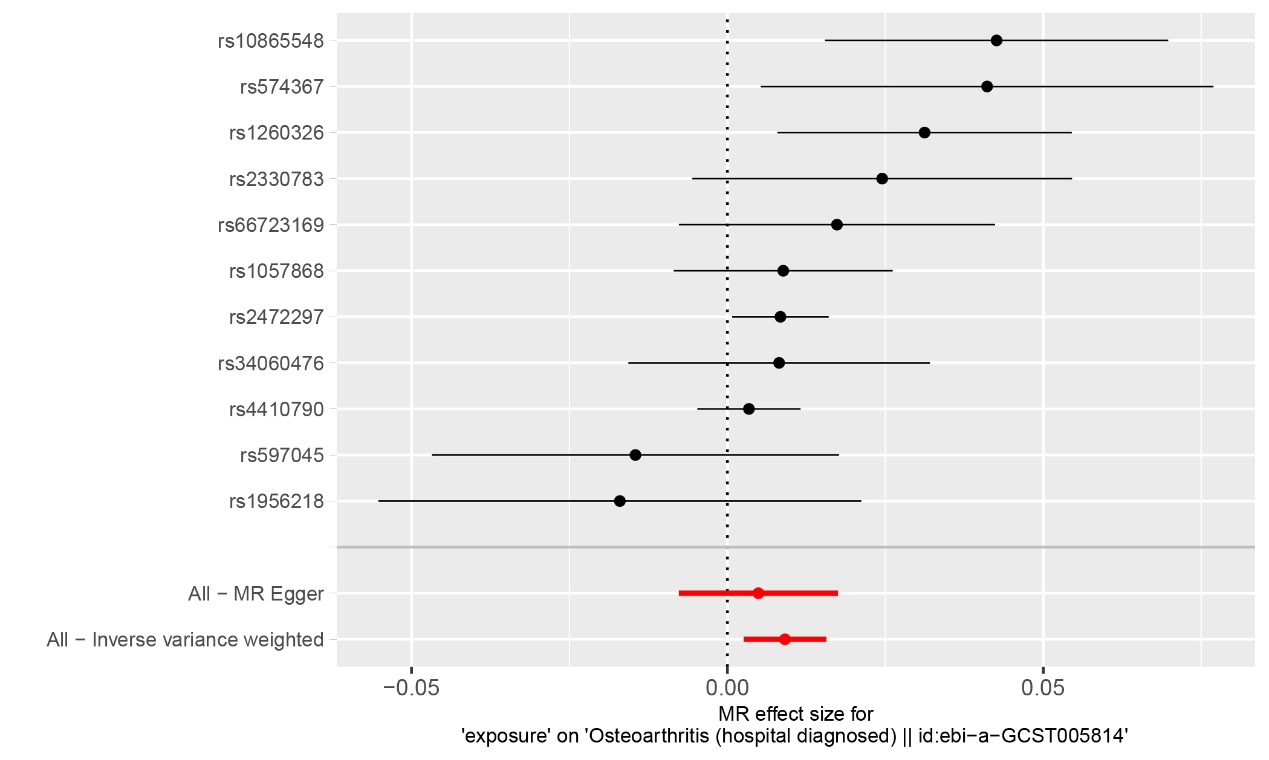
**

**Figure 1S.** The forest plot for the causal effects of coffee consumption-associated SNPs on total OA using primary genetic instruments.

**
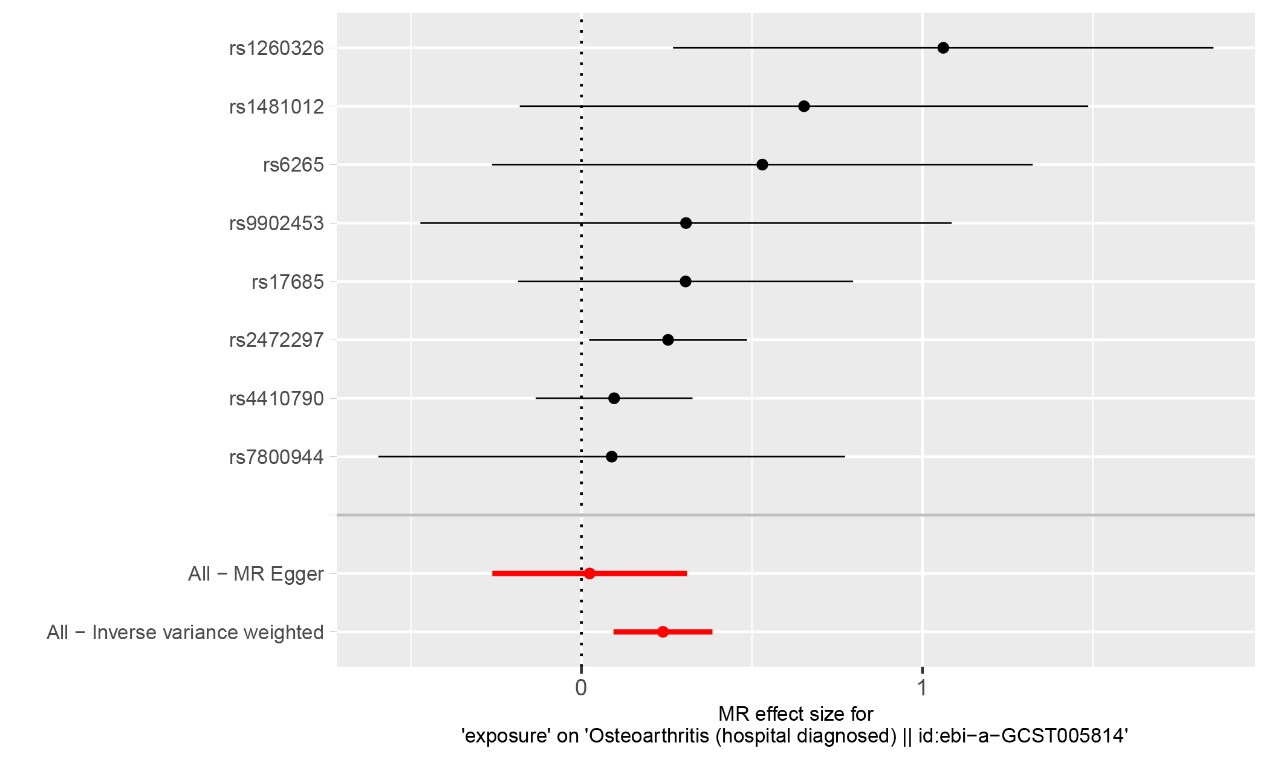
**

**Figure 2S.** The forest plot for the causal effects of coffee consumption-associated SNPs on total OA using secondary genetic instruments.

**
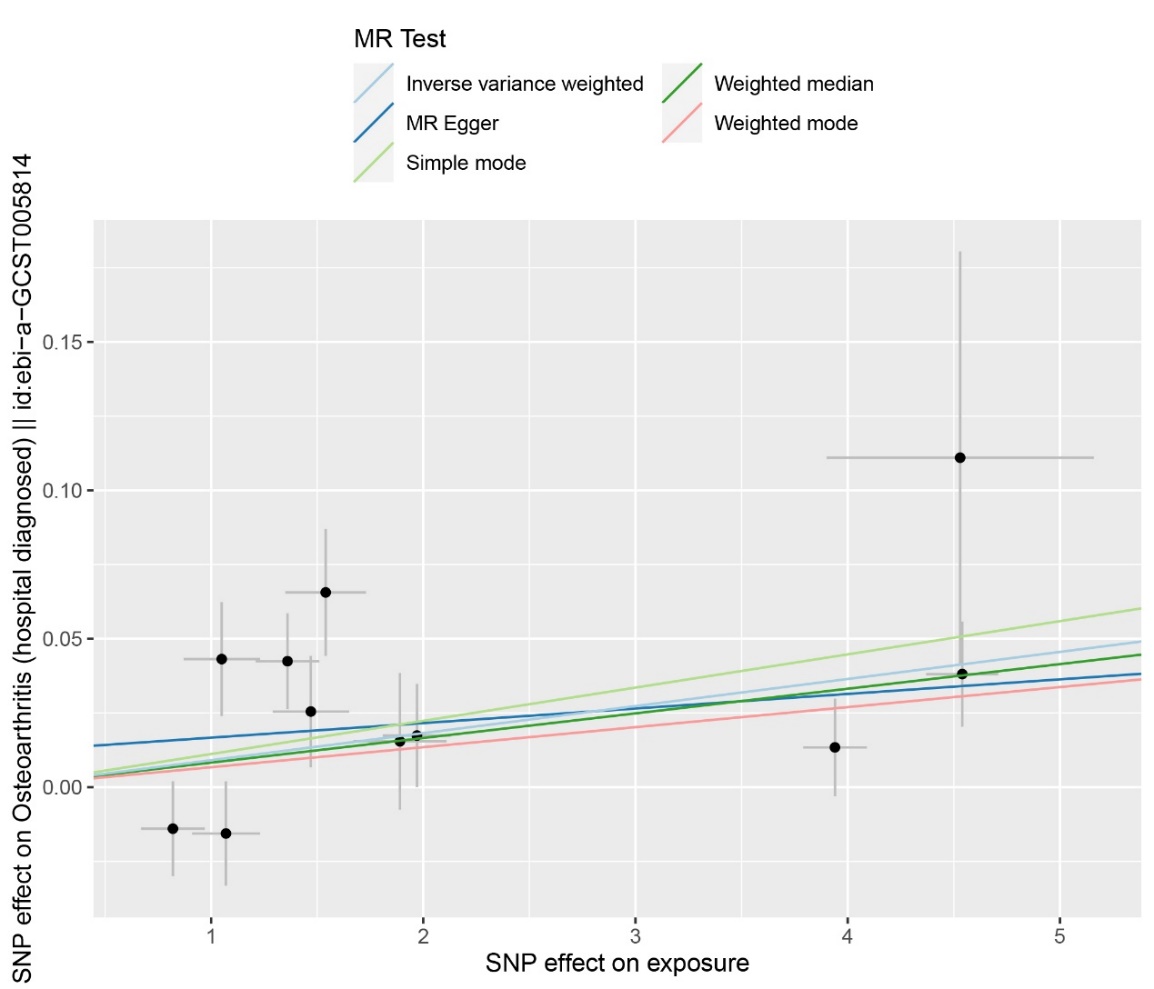
**

**Figure 3S.** Scatter plot of genetic associations comparing coffee consumption to the genetic associations with total OA using primary genetic instruments.


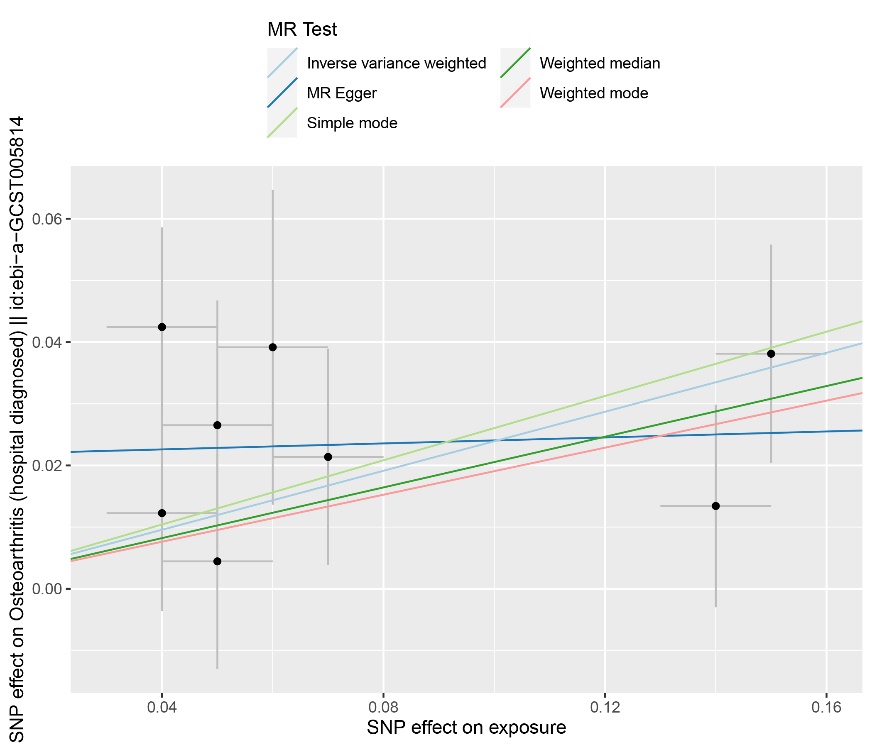


**Figure 4S.** Scatter plot of genetic associations comparing coffee consumption to the genetic associations with total OA using secondary genetic instruments.


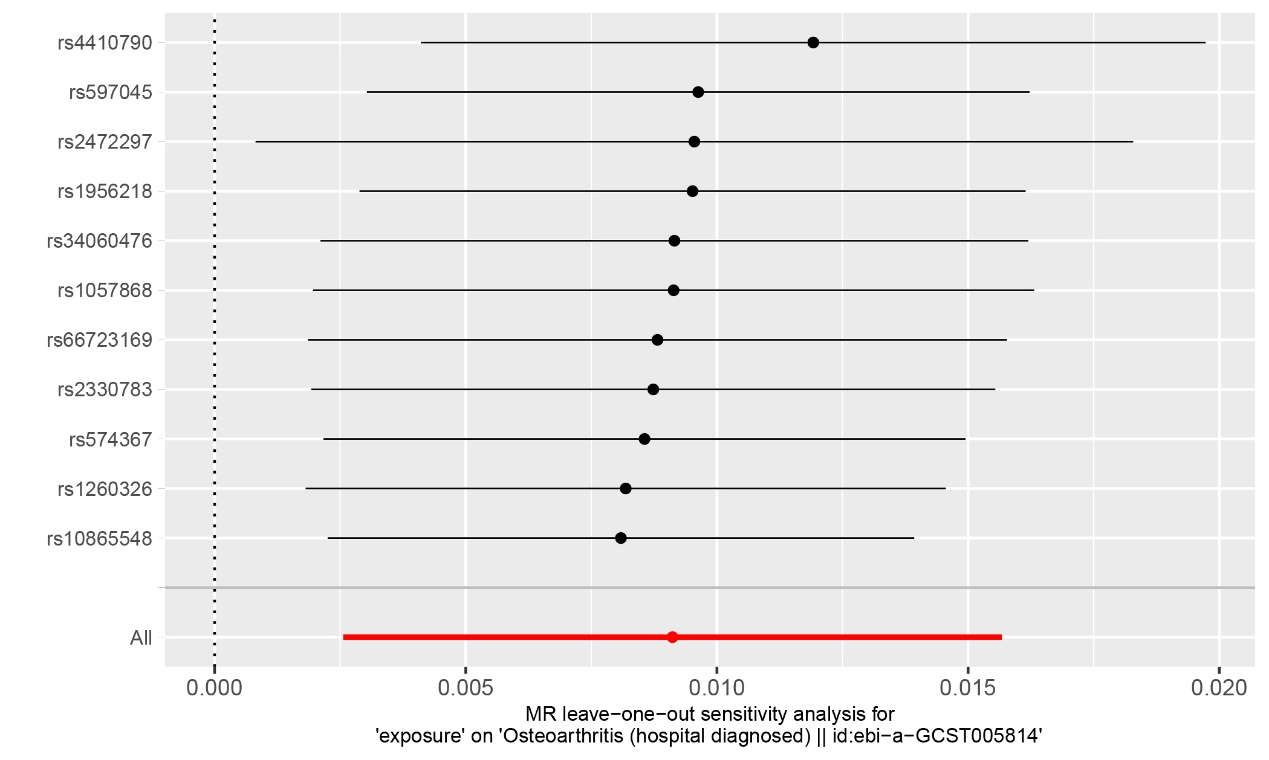


**Figure 5S.** Leave-one-out sensitivity analysis for the total OA using primary genetic instruments


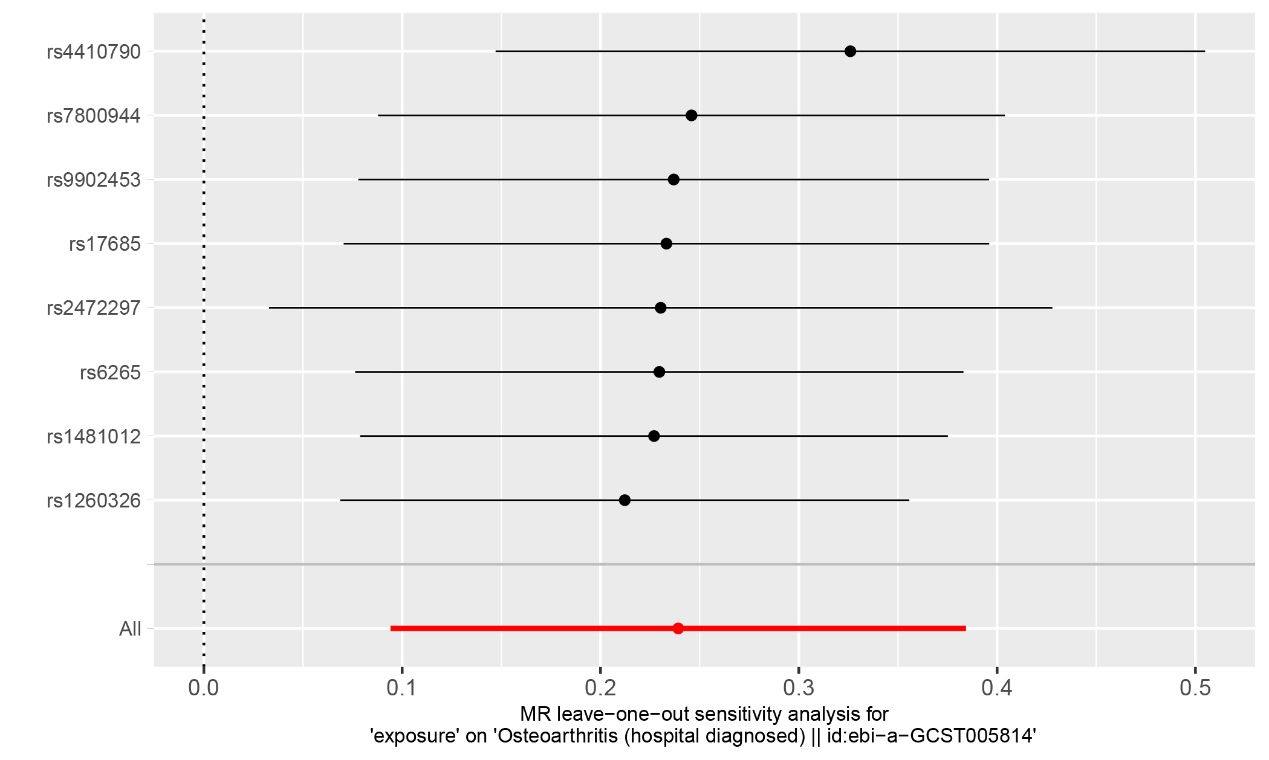


**Figure 6S.** Leave-one-out sensitivity analysis for the total OA using secondary genetic instruments


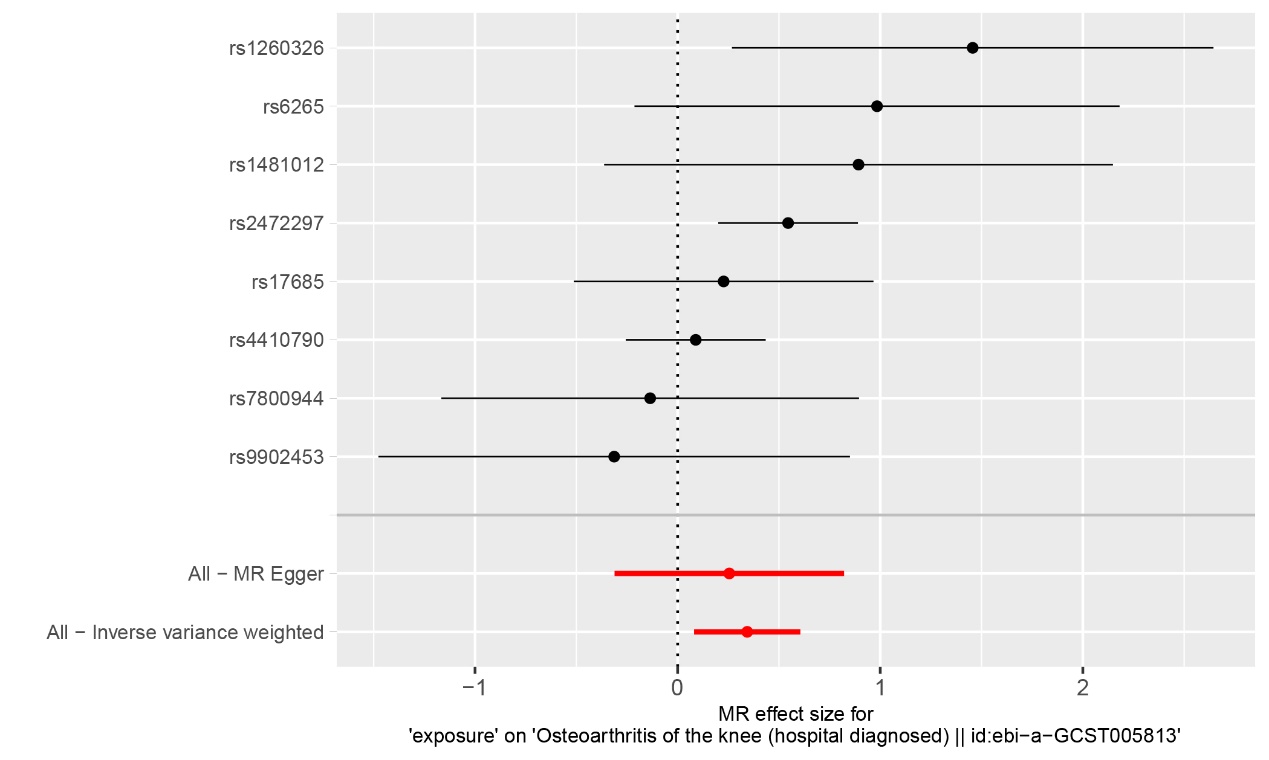


**Figure 7S.** The forest plot for the causal effects of coffee consumption-associated SNPs on knee OA using primary genetic instruments.


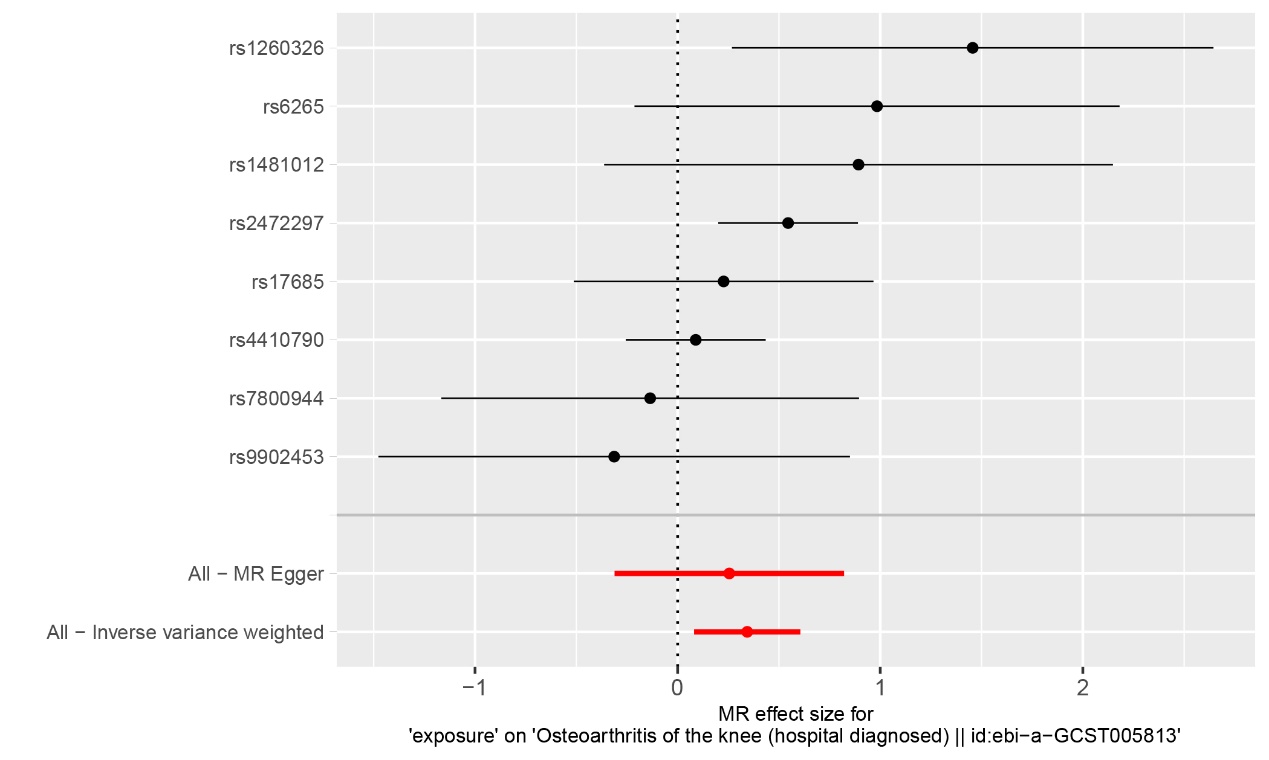


**Figure 8S.** The forest plot for the causal effects of coffee consumption-associated SNPs on knee OA using secondary genetic instruments.


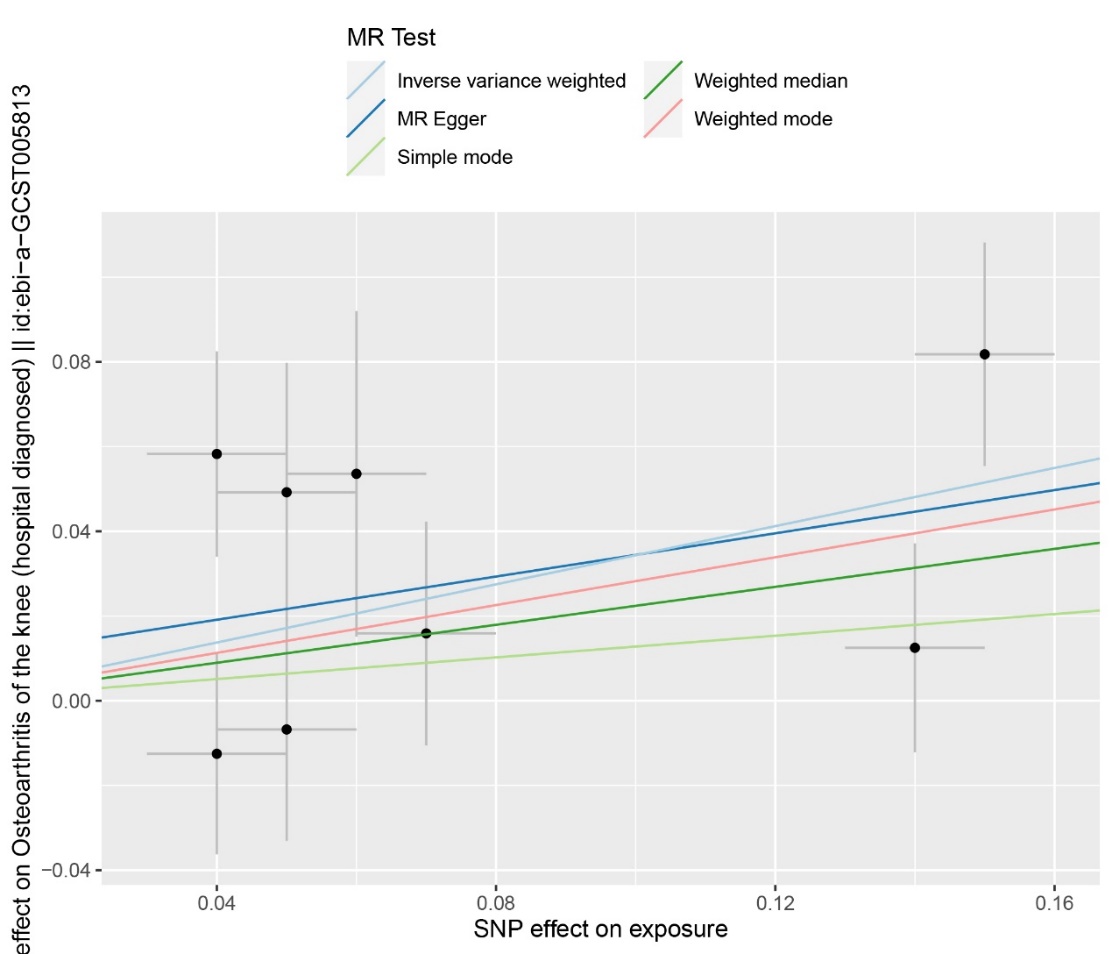


**Figure 9S.** Scatter plot of genetic associations comparing coffee consumption to the genetic associations with knee OA using primary genetic instruments.


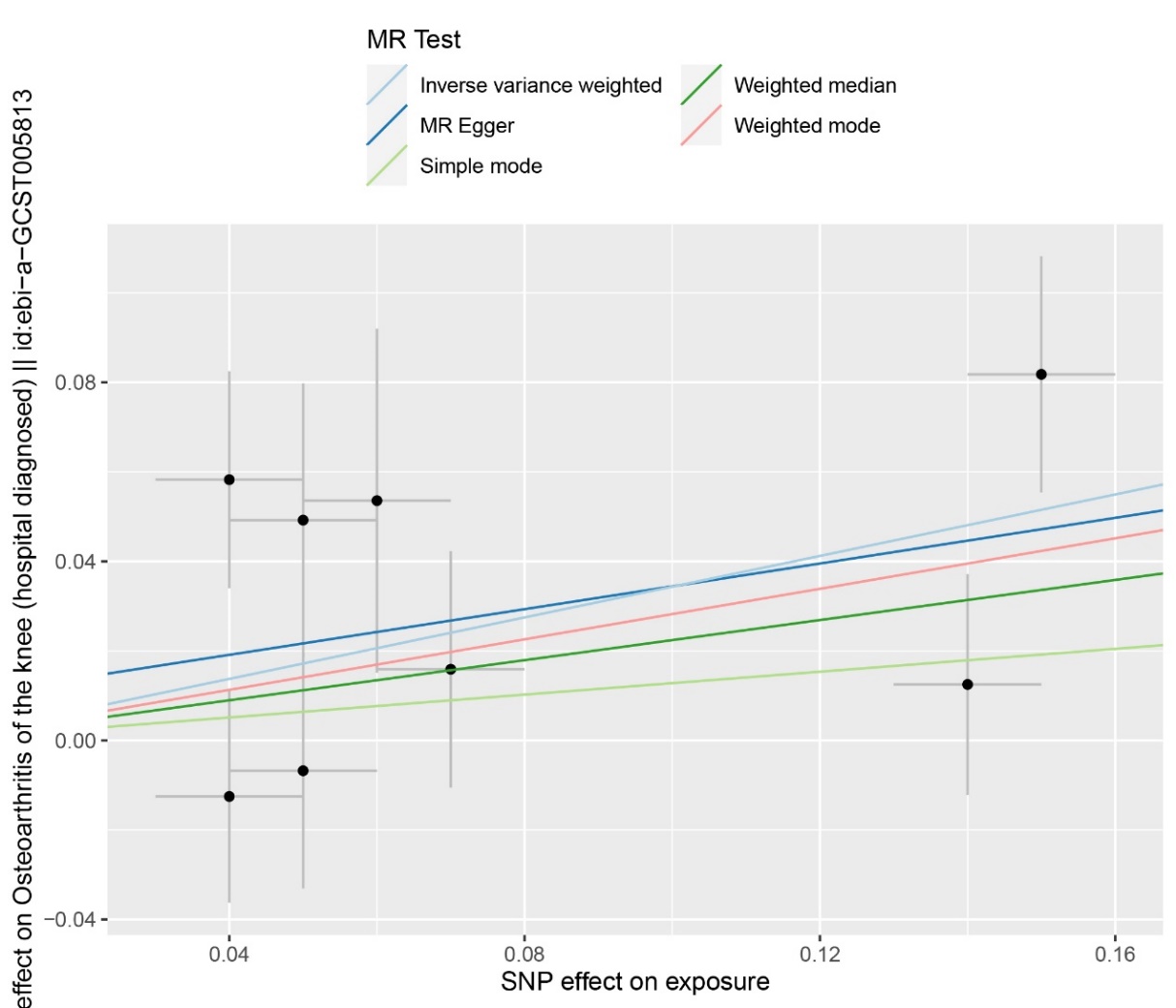


**Figure 10S.** Scatter plot of genetic associations comparing coffee consumption to the genetic associations with knee OA using secondary genetic instruments.


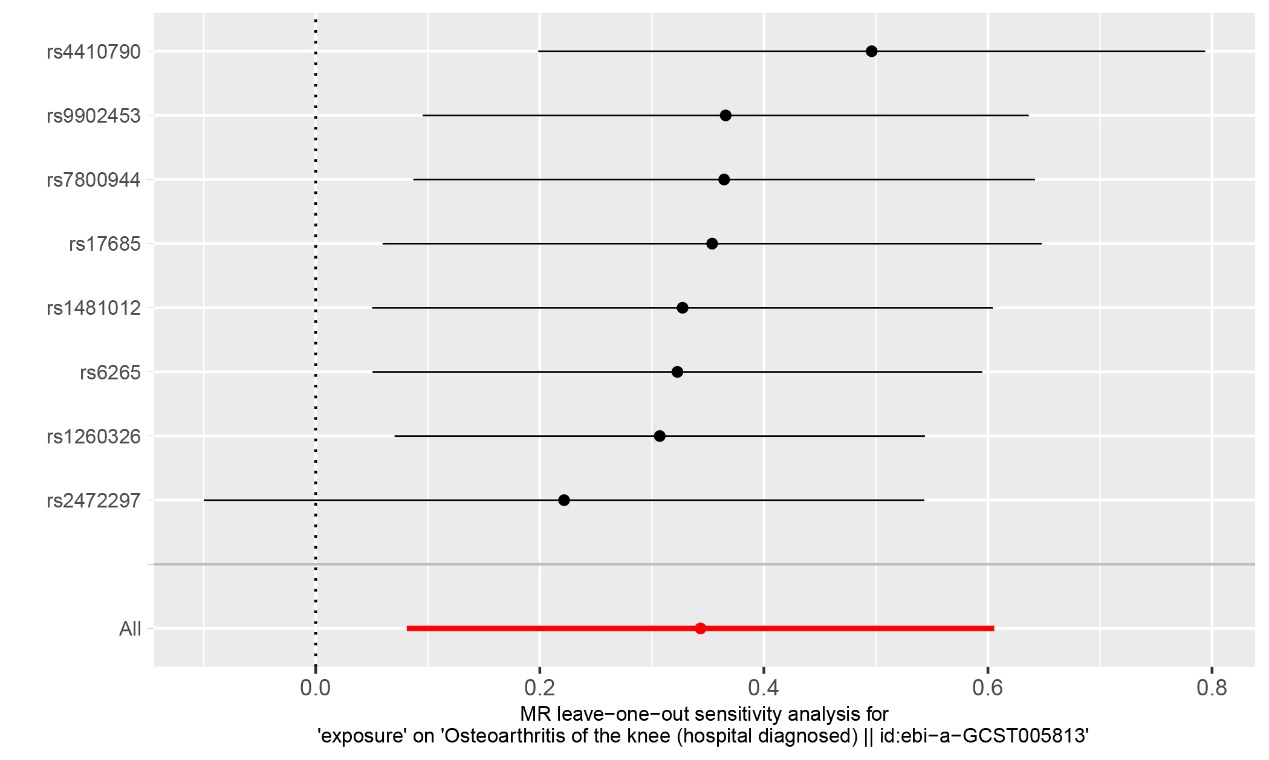


**Figure 11S.** Leave-one-out sensitivity analysis for the knee OA using primary genetic instruments


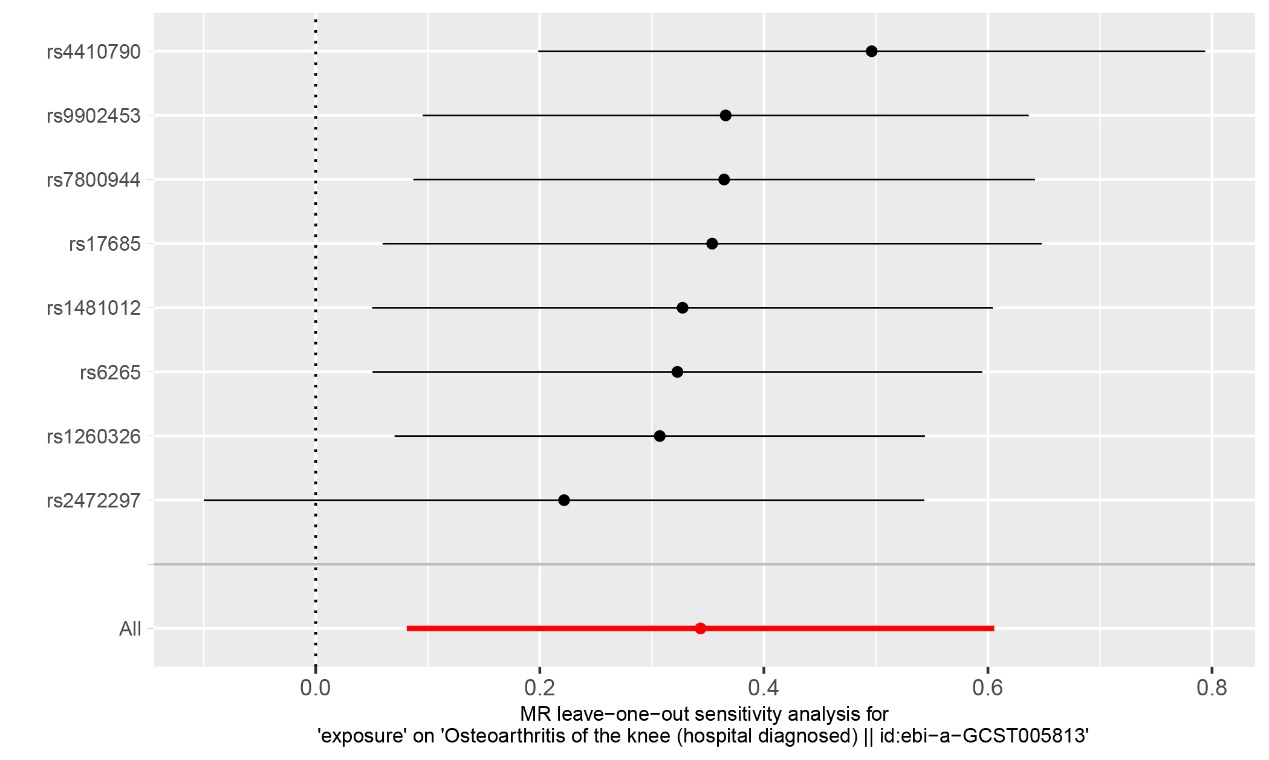


**Figure 12S.** Leave-one-out sensitivity analysis for the knee OA using secondary genetic instruments


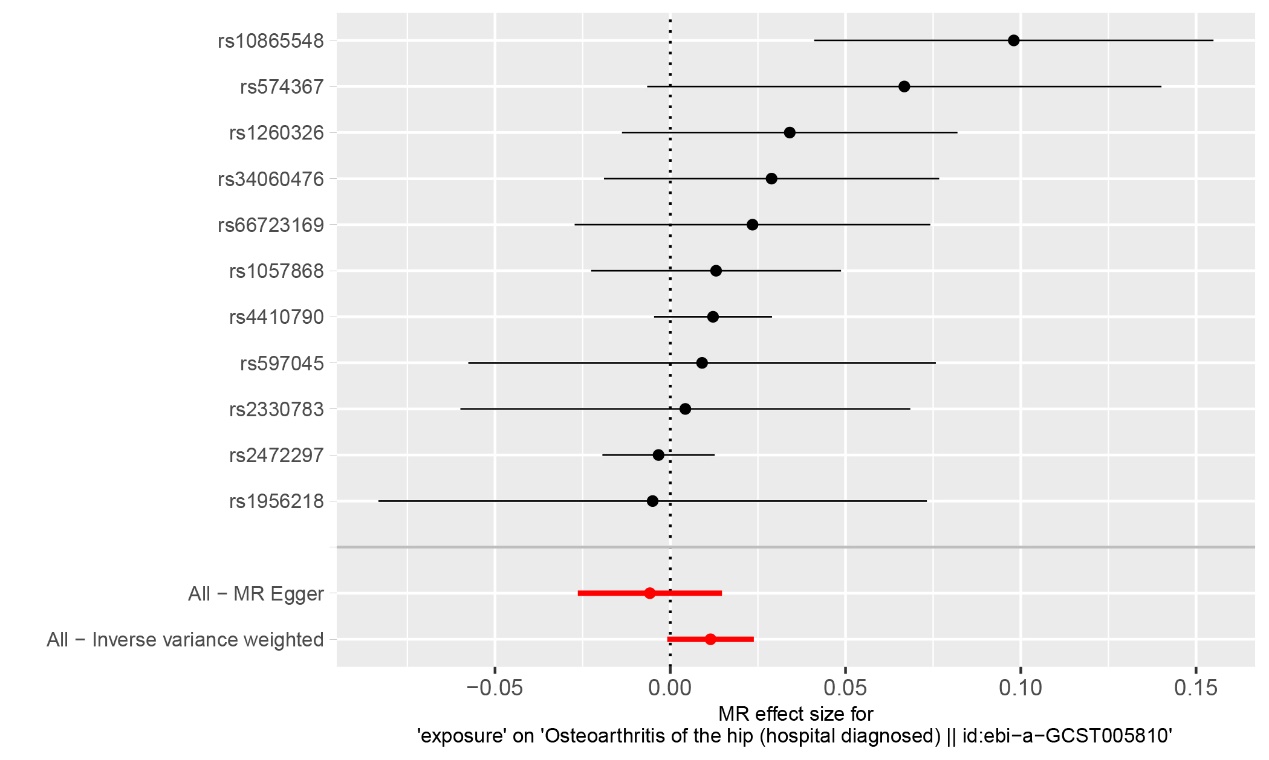


**Figure 13S.** The forest plot for the causal effects of coffee consumption-associated SNPs on hip OA using primary genetic instruments.


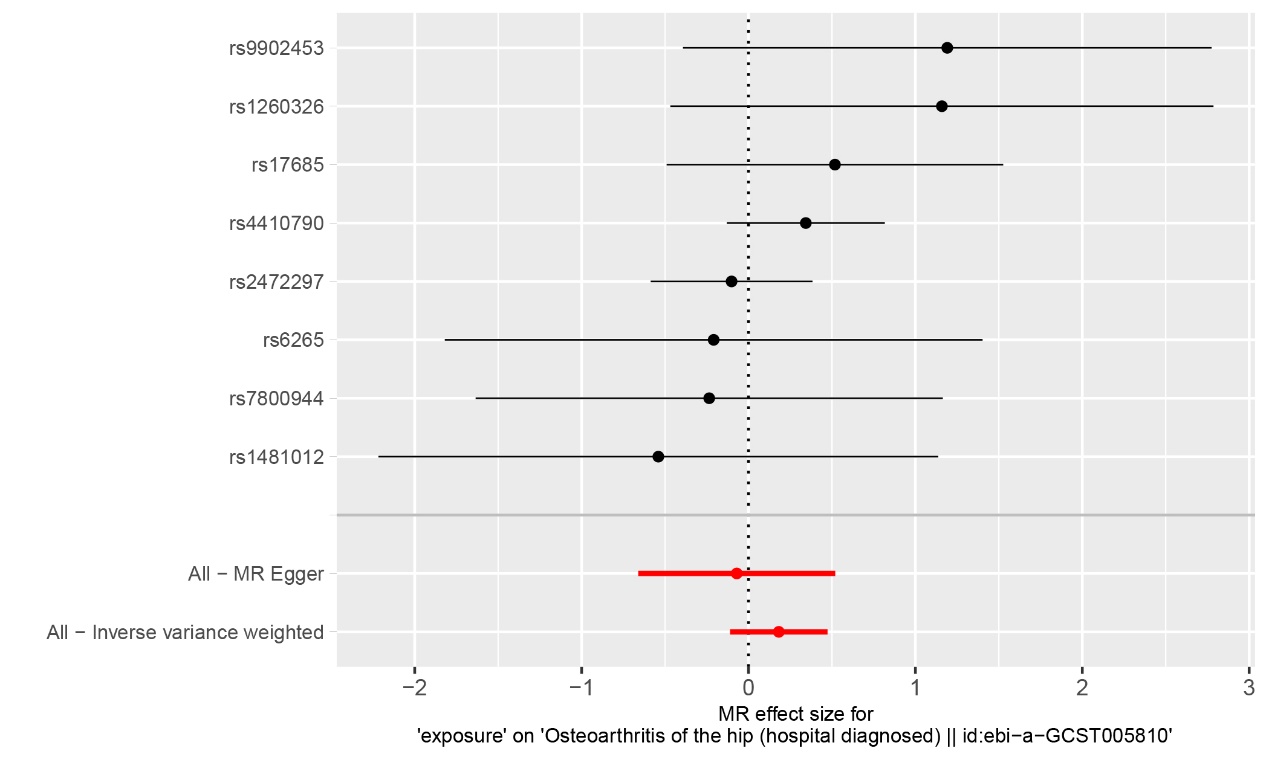


**Figure 14S.** The forest plot for the causal effects of coffee consumption-associated SNPs on hip OA using secondary genetic instruments.


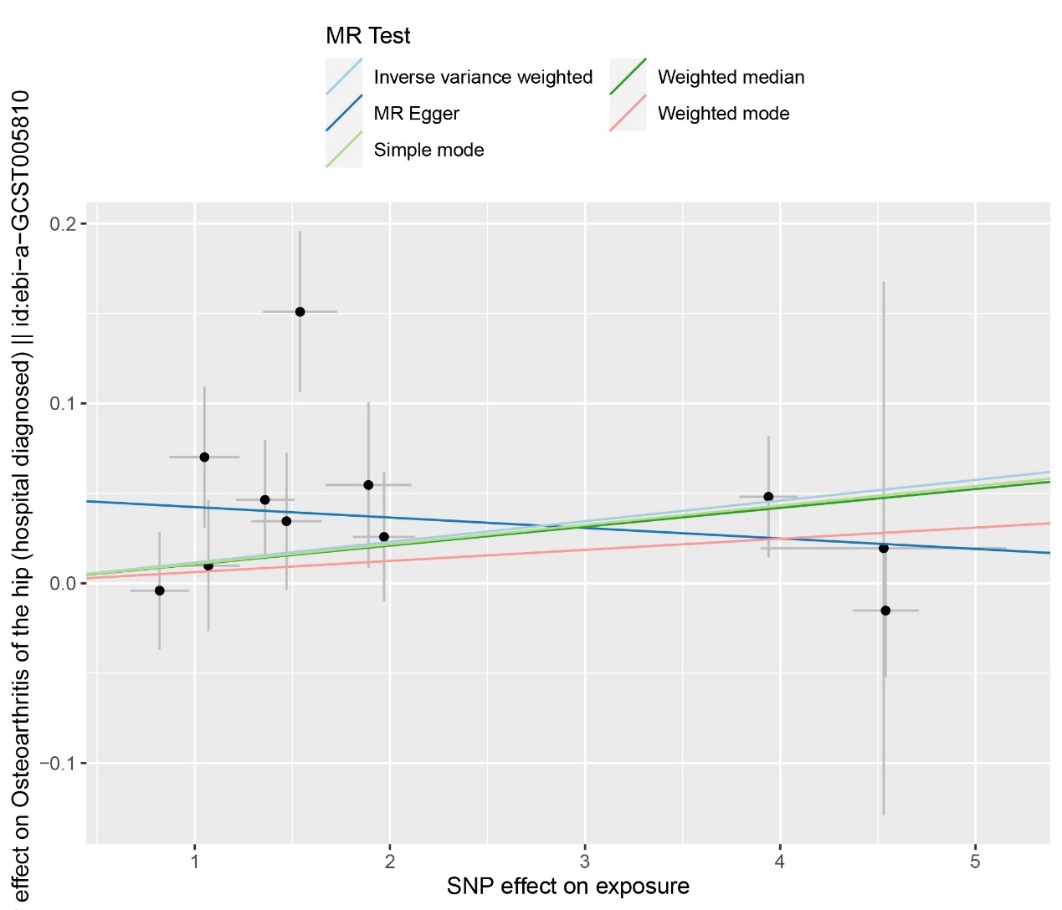


**Figure 15S.** Scatter plot of genetic associations comparing coffee consumption to the genetic associations with hip OA using primary genetic instruments.


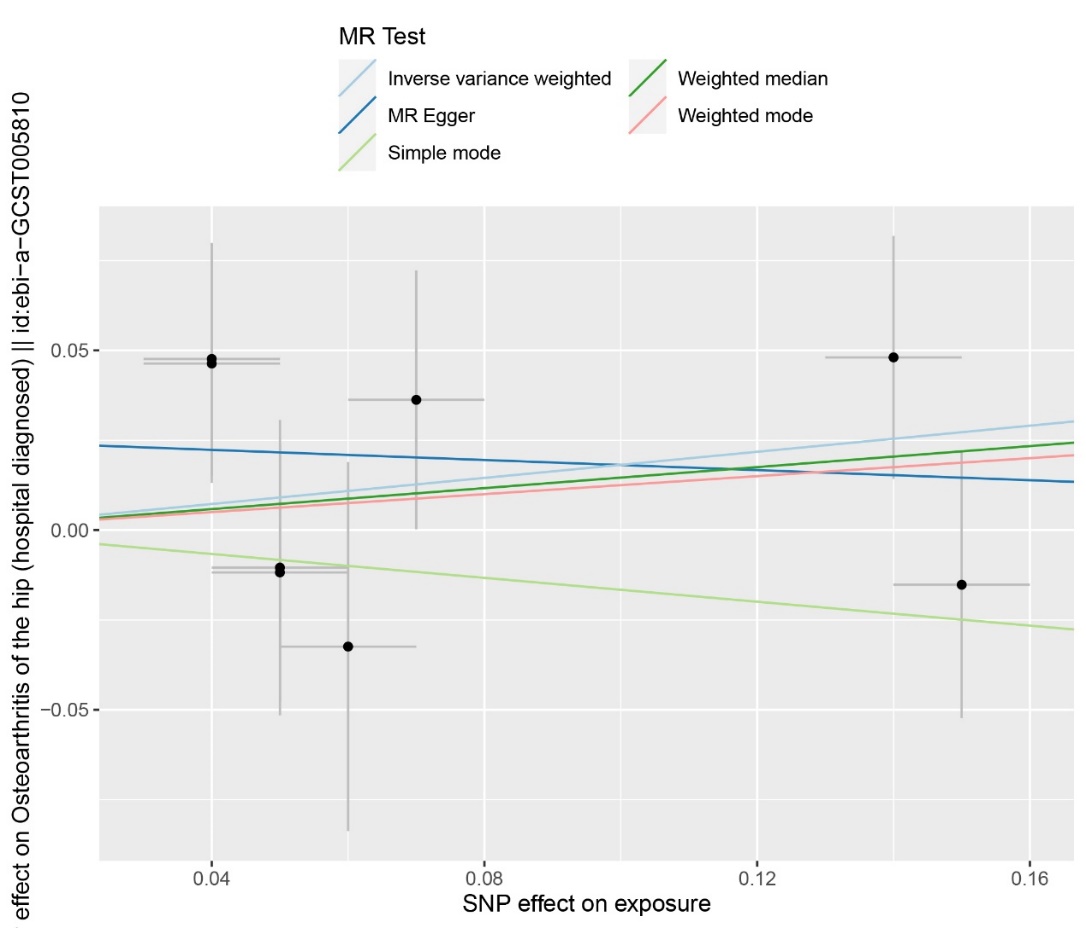


**Figure 16S.** Scatter plot of genetic associations comparing coffee consumption to the genetic associations with hip OA using secondary genetic instruments.


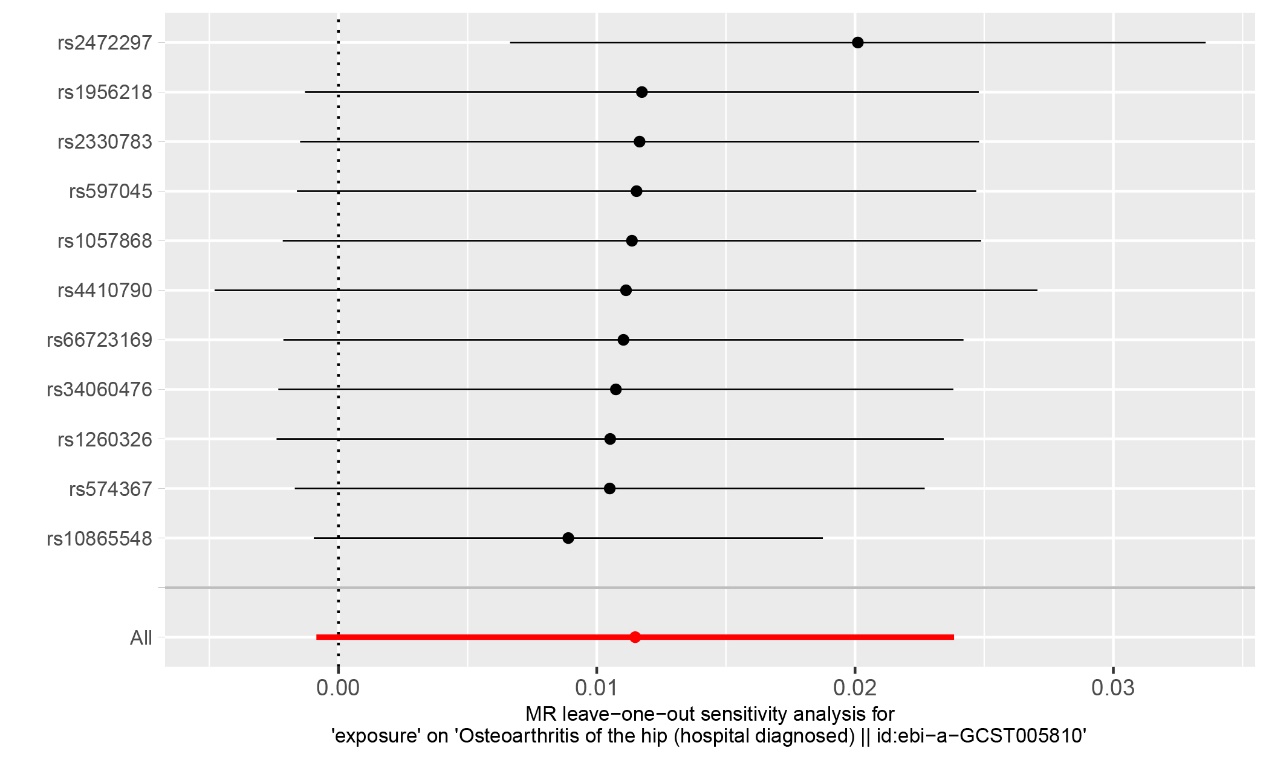


**Figure 17S.** Leave-one-out sensitivity analysis for the hip OA using primary genetic instruments

**
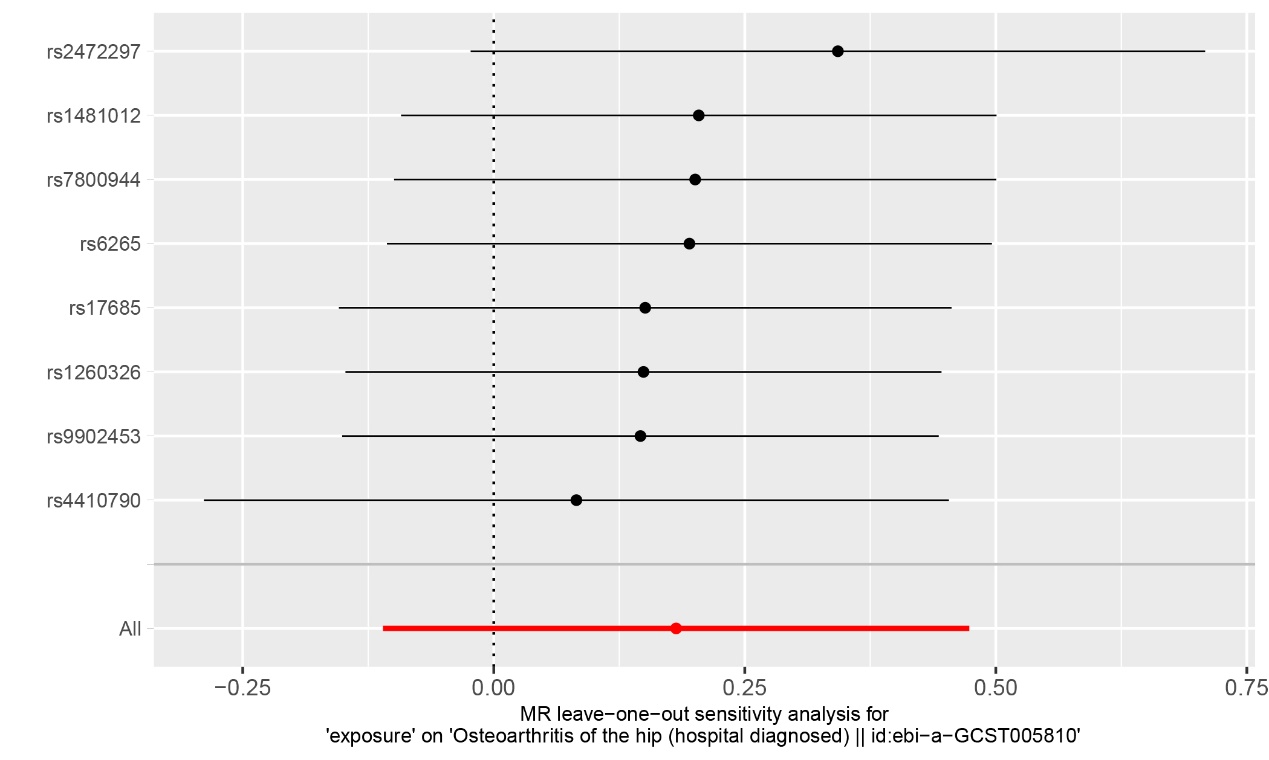
**

**Figure 18S.** Leave-one-out sensitivity analysis for the hip OA using secondary genetic instruments


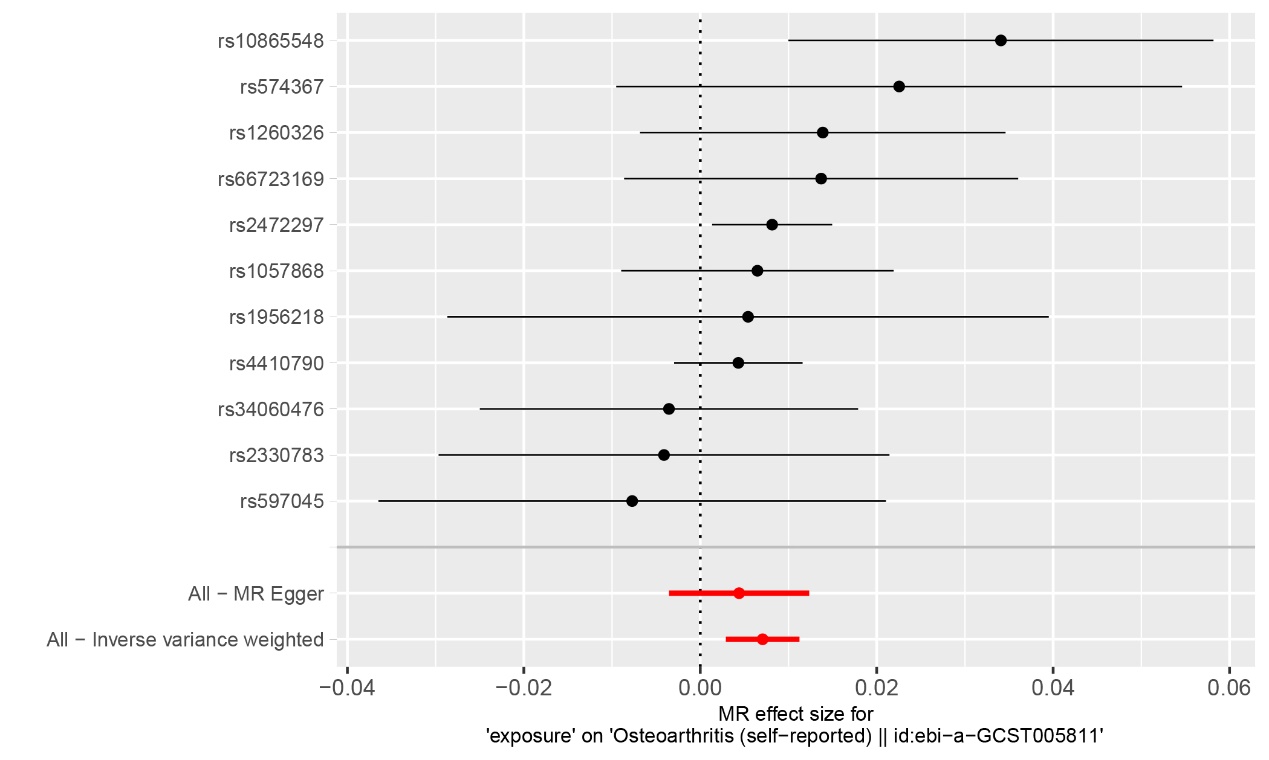


**Figure 19S.** The forest plot for the causal effects of coffee consumption-associated SNPs on self-reported OA using primary genetic instruments.


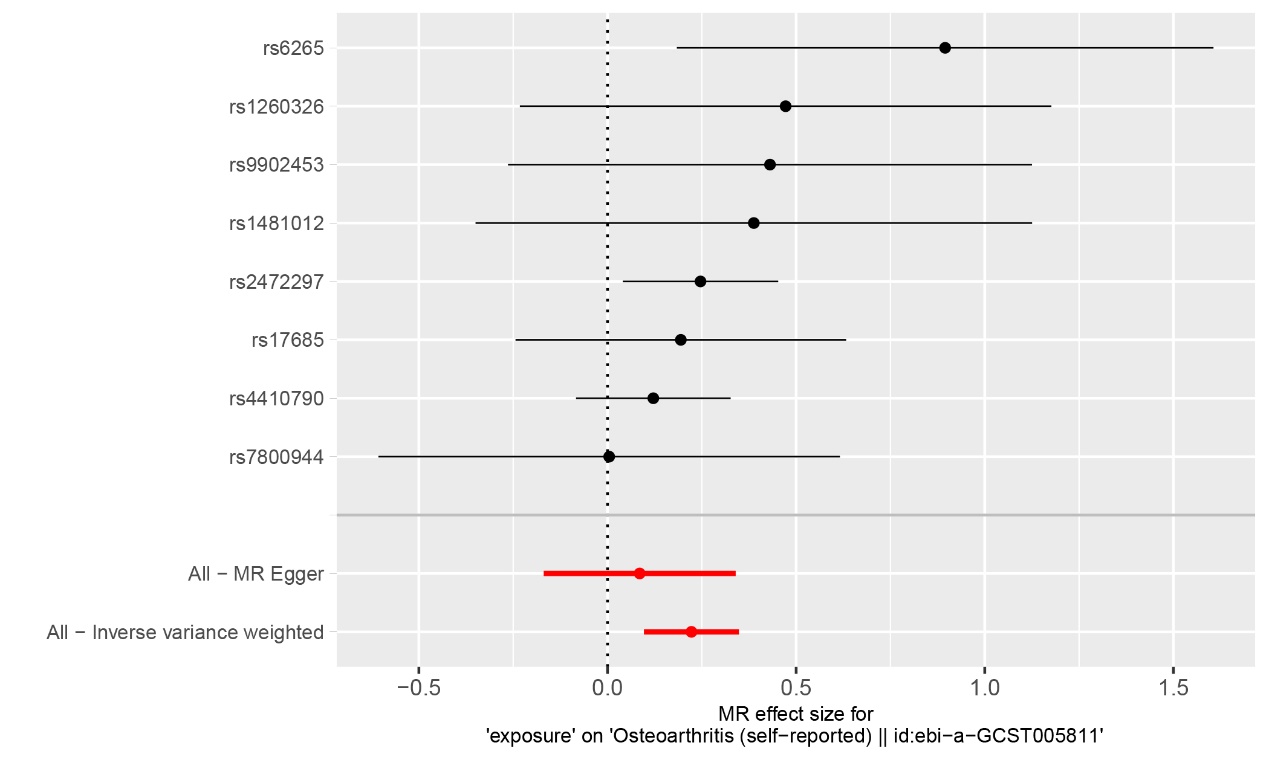


**Figure 20S.** The forest plot for the causal effects of coffee consumption-associated SNPs on self-reported OA using secondary genetic instruments.


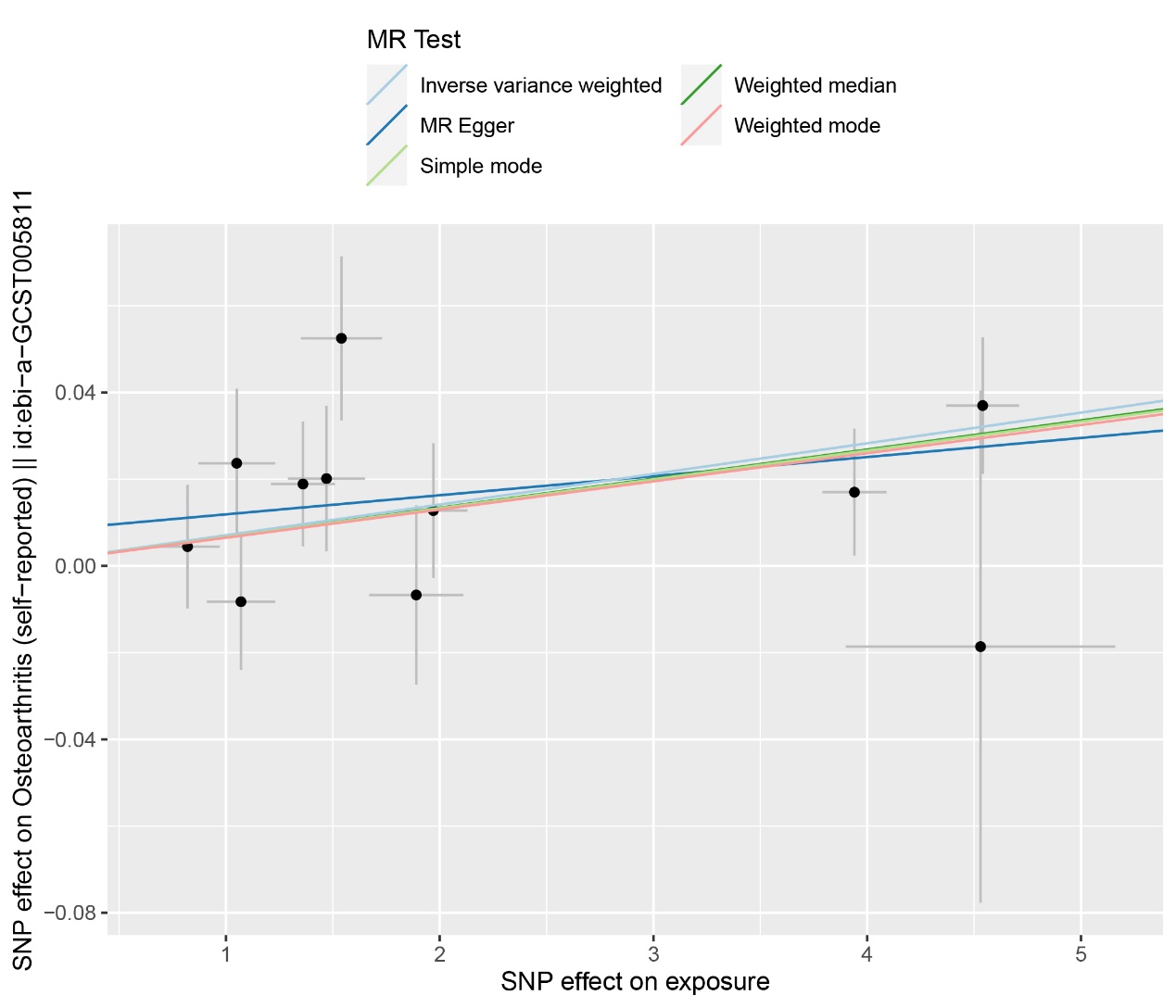


**Figure 21S.** Scatter plot of genetic associations comparing coffee consumption to the genetic associations with self-reported OA using primary genetic instruments.


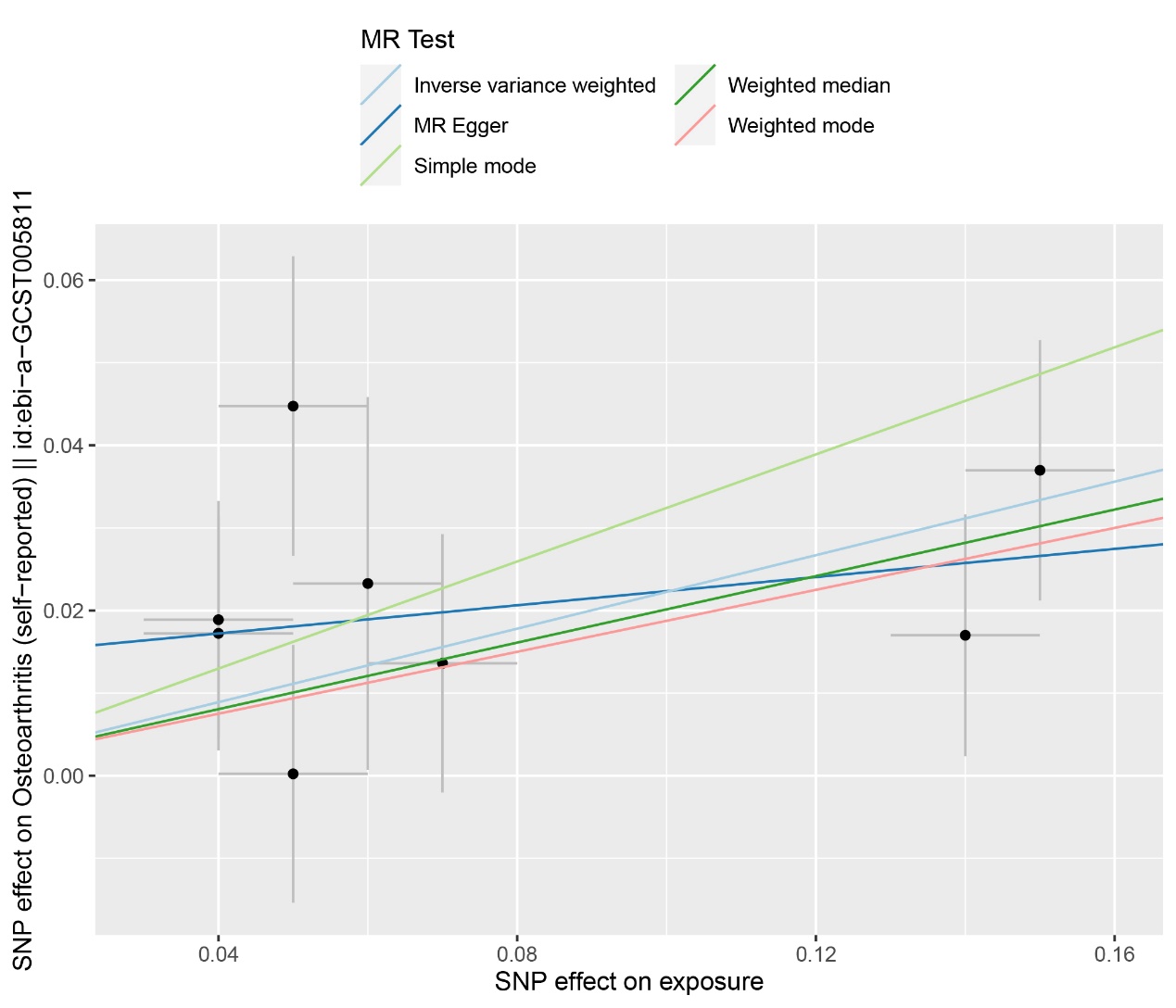


**Figure 22S.** Scatter plot of genetic associations comparing coffee consumption to the genetic associations with self-reported OA using secondary genetic instruments.


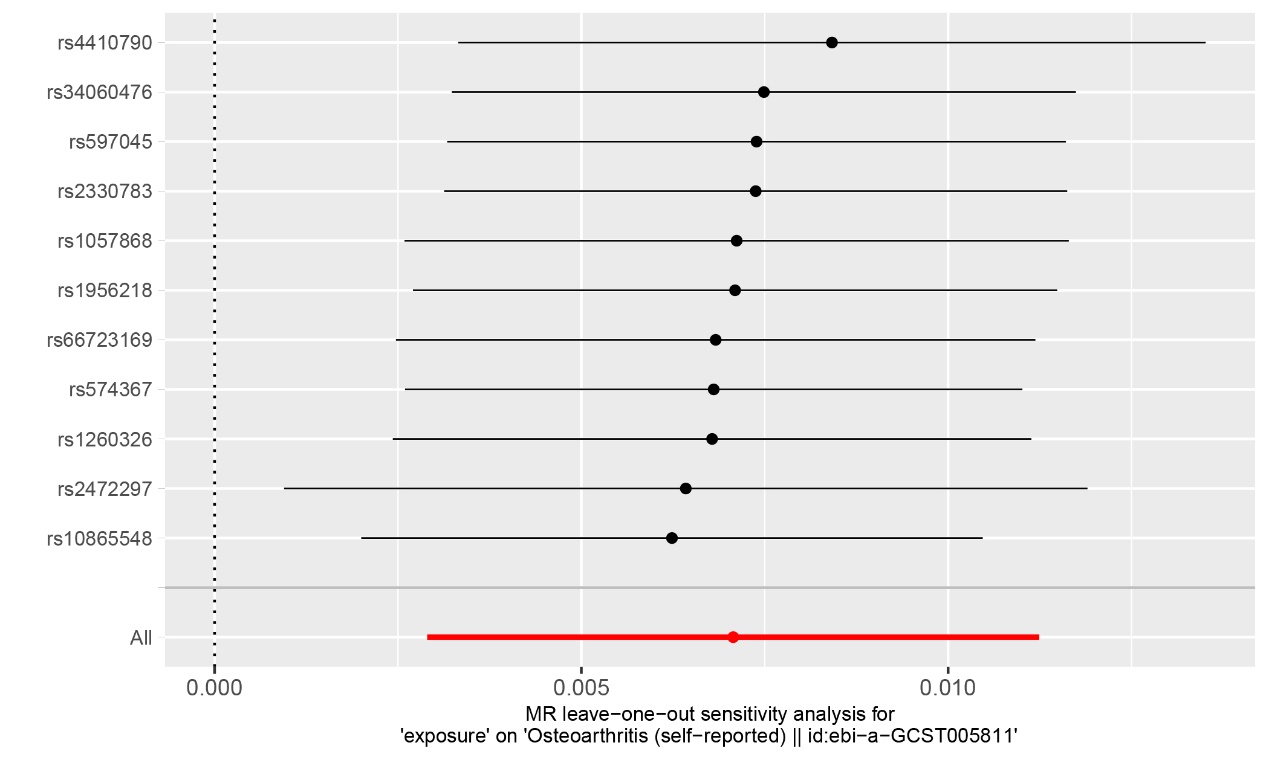


**Figure 23S.** Leave-one-out sensitivity analysis for the self-reported OA using primary genetic instruments


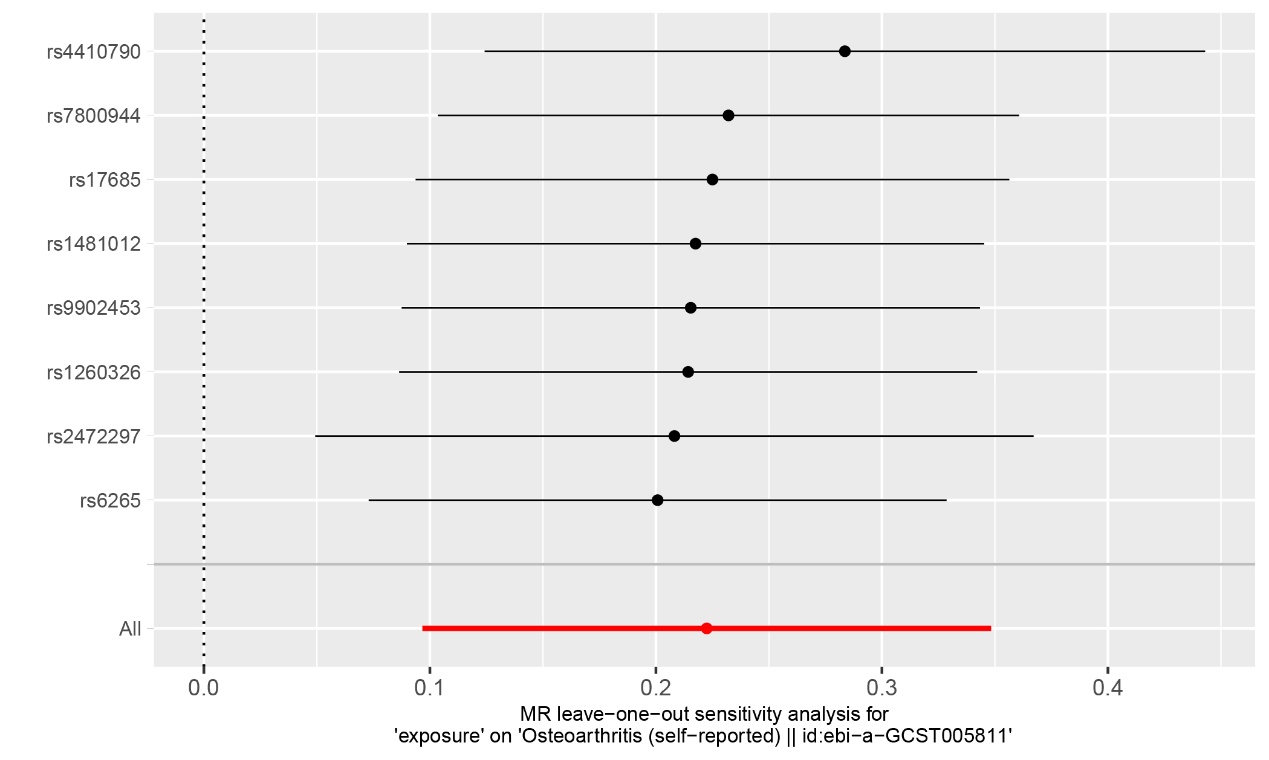


**Figure 24S.** Leave-one-out sensitivity analysis for the self-reported OA using secondary genetic instruments
